# Short exposure to photo-oxidative damage triggers molecular signals indicative of early retinal degeneration

**DOI:** 10.3389/fimmu.2023.1088654

**Published:** 2023-04-27

**Authors:** Yvette Wooff, Adrian V. Cioanca, Elly Wills, Joshua A. Chu-Tan, Rakshanya Sekar, Riccardo Natoli

**Affiliations:** ^1^ Clear Vision Research Group, Eccles Institute of Neuroscience, John Curtin School of Medical Research, College of Health and Medicine, The Australian National University, Acton, ACT, Australia; ^2^ School of Medicine and Psychology, College of Health and Medicine, The Australian National University, Acton, ACT, Australia

**Keywords:** retina, retinal degeneration, age-related macular degeneration, early-stage age-related macular degeneration, rodent model, diagnostic biomarkers, inflammation, microRNA

## Abstract

**Introduction:**

Age-related macular degeneration (AMD) is the leading cause of blindness in the developed world, currently affecting over 350 billion people globally. For the most prevalent late-stage form of this disease, atrophic AMD, there are no available prevention strategies or treatments, in part due to inherent difficulties in early-stage diagnosis. Photo-oxidative damage is a well-established model for studying inflammatory and cell death features that occur in late-stage atrophic AMD, however to date has not been investigated as a potential model for studying early features of disease onset. Therefore, in this study we aimed to determine if short exposure to photo-oxidative damage could be used to induce early retinal molecular changes and advance this as a potential model for studying early-stage AMD.

**Methods:**

C57BL/6J mice were exposed to 1, 3, 6, 12, or 24h photo-oxidative damage (PD) using 100k lux bright white light. Mice were compared to dim-reared (DR) healthy controls as well as mice which had undergone long periods of photo-oxidative damage (3d and 5d-PD) as known timepoints for inducing late-stage retinal degeneration pathologies. Cell death and retinal inflammation were measured using immunohistochemistry and qRT-PCR. To identify retinal molecular changes, retinal lysates were sent for RNA sequencing, following which bioinformatics analyses including differential expression and pathway analyses were performed. Finally, to investigate modulations in gene regulation as a consequence of degeneration, microRNA (miRNA) expression patterns were quantified using qRT-PCR and visualized using *in situ* hybridization.

**Results:**

Short exposure to photo-oxidative damage (1-24h-PD) induced early molecular changes in the retina, with progressive downregulation of homeostatic pathways including metabolism, transport and phototransduction observed across this time-course. Inflammatory pathway upregulation was observed from 3h-PD, preceding observable levels of microglia/macrophage activation which was noted from 6h-PD, as well as significant photoreceptor row loss from 24h-PD. Further rapid and dynamic movement of inflammatory regulator miRNA, miR-124-3p and miR-155-5p, was visualized in the retina in response to degeneration.

**Conclusion:**

These results support the use of short exposure to photo-oxidative damage as a model of early AMD and suggest that early inflammatory changes in the retina may contribute to pathological features of AMD progression including immune cell activation and photoreceptor cell death. We suggest that early intervention of these inflammatory pathways by targeting miRNA such as miR-124-3p and miR-155-5p or their target genes may prevent progression into late-stage pathology.

## Introduction

Age-related macular degeneration (AMD) is a chronic inflammatory disease of the retina, characterized by the progressive death of the light sensing photoreceptor cells and the underlying retinal pigmented epithelium (RPE), resulting in permanent, irreversible blindness (1, 2). Currently AMD affects 1:7 people over the age of 50, and for the more prevalent late-stage form of this disease, atrophic AMD, there are no available treatments (3, 4). While AMD advancement is slow, and progresses through early, intermediate, and late-stages (1); early diagnostic tests are only predictive and are limited in sensitivity and reliability, meaning that AMD is often not detected until late-stage (5–7). Therefore, with no treatment options or available cure, there is an imperative need to understand molecular changes which occur early in retinal degenerations, and how these may contribute to disease progression (8).

Molecular markers detected in the serum, plasma or ocular fluids are widely investigated as useful diagnostic and prognostic markers for AMD (9–11), however still rely on their association with observable retinal pathologies such as drusen deposition (yellow lipid accumulation), visual impairment, and areas of cell death in the central retina (12). Furthermore, without a direct relationship to molecular changes occurring within the retina, systemic profiling can only be used as a companion diagnostic, due to its strongly correlative nature. Therefore, to truly gain diagnostic power, it is necessary to identify and define early molecular changes that occur within the retina, in particular, those which precede the onset of early-stage AMD pathologies, and in particular photoreceptor cell death. As there are limited clinical markers to predict the onset of early AMD, the use of rodent models of retinal degeneration which mimic key features of AMD is essential to investigate and determine early molecular changes occurring in the degenerating retina.

Light-induced oxidative stress is a known risk factor for AMD, given the chronic exposure and build-up of natural reaction oxygen species by-products produced during phototransduction (2, 13, 14). Therefore, photo-oxidative damage has been used as a well-established model of retinal degeneration, employing prolonged periods (days to weeks) of bright-white light to induce pathological features of AMD including focal photoreceptor and RPE cell death, a progressively expanding central lesion, upregulated oxidative stress and inflammatory pathways, and recruitment and activation of microglia/macrophage immune cells (15–18). While photo-oxidative damage along with genetic or chemically induced models of retinal degeneration are routinely used to investigate causative factors and therapeutic strategies to treat AMD (15, 16, 19–23), to date little progress has been made in charactering early diagnostic markers. Given that currently characterized features of these models; in particular, the onset of photoreceptor cell death, are regarded as late-stage manifestations of disease, investigating time periods prior to measurable levels of cell death or vision loss is required to distinguish early molecular markers of disease onset and progression. Therefore, in this work we sought to investigate if short periods of photo-oxidative damage (1h-24h) could be used to elicit measurable changes in the molecular profile of the retina, which could be linked to progressive degeneration, and be used as early diagnostic markers and potential early intervention/treatment options for AMD.

Key findings from this work have demonstrated that early molecular changes can be detected in the retina prior to observed degeneration using short-term photo-oxidative damage and have characterized inflammatory genes and pathways unique to early degeneration. Furthermore, this work has identified that key microRNA (miRNA), miR-124-3p and miR-155-5p known to be involved in late-stage AMD (23–25), may play an important role in regulating the early degenerative response, and therefore represent ideal therapeutic targets for slowing the progression of degeneration.

## Methods

### Animal paradigms

#### Animal handling

All experiments were conducted at the ANU in accordance with the ARVO Statement for the Use of Animals in Ophthalmic and Vision Research and with approval from the Australian National University’s (ANU) Animal Experimentation Ethics Committee (AEEC) (Ethics ID: A2020/41; Rodent models and treatments for retinal degenerations). Adult male and female C57BL/6J wild-type (WT) mice (aged 60 postnatal days; (P50) at experimental onset, N=12 histology, N=4 sequencing) were purchased and transported in temperature and light-controlled conditions with enrichment, food, and water, from Australian BioResources (ABR), (Garvan Institute of Medical Research, New South Wales (NSW)). Mice were acclimated for one week at the ANU according to AEEC requirements before experimentation. Mice were bred, reared, transported, and housed under 12 h light (5 lux)/dark cycle conditions with free access to food and water.

#### Photo-oxidative damage

Mice were subjected to photo-oxidative damage (PD) for up to 5 days (1h, 3h, 6h, 12h, 24h, 3 days and 5 days PD) as described previously (16). Briefly, mice were placed into Perspex boxes coated with a reflective interior surface and exposed to 100 K lux white light from light-emitting diodes (LED). Animals were administered pupil dilator (Minims^®^ atropine sulphate 1% w/v; Bausch and Lomb) to both eyes twice a day (9am and 4pm) during the course of the damage paradigm. Following photo-oxidative damage, retinal morphology was assessed and compared between photo-oxidative damage and dim-reared (DR; 5 lux light) controls.

### Retinal tissue analysis

#### Tissue collection and preparation

Animals were ethically euthanized with CO_2_ following PD. The superior surface of the left eye was marked and enucleated, then immersed in 4% paraformaldehyde for 3 hours at 4°C. Eyes were then cryopreserved in 15% sucrose solution at 4°C overnight, embedded in OCT medium (Tissue Tek, Sakura, Japan) and cryosectioned at 12 μm in a parasagittal plane (superior to inferior) using a CM 1850 Cryostat (Leica Biosystems, Germany). To ensure accurate comparisons were made for histological analysis, only sections containing the optic nerve head were used for analysis. The retina from the right eye was excised through a corneal incision and placed into RNAlater solution (Thermo Fisher Scientific, MA, United States) at 4°C overnight and then stored at −80°C until further use.

#### Immunolabelling

Immunohistochemical analysis of retinal cryosections was performed as previously described (26). Fluorescence was visualized and images taken using a laser-scanning A1^+^ confocal microscope at 20 and 40x magnification (Nikon, Tokyo, Japan). Images panels were analyzed using ImageJ V2.0 software and assembled using Illustrator software (Adobe Systems, CA, United States).

#### Immunohistochemistry and analysis

Immunolabelling for Ionized Calcium Binding Adaptor molecule 1 IBA1 (1:500, 019-19741, Wako, Osaka, Japan), a marker of microglia and macrophage immune cells and Glial Fibrillary Acidic Protein (GFAP) (1:500, ASTR06, MA5-12023, Invitrogen, Massachusetts, USA), a marker of glial cell stress was performed as previously described (26). Retinal cryosections were stained with the DNA-specific dye bisbenzimide (BBZ; 1:10000, Sigma-Aldrich, MO, United States) to visualize the cellular layers. The number and morphology (ramified *vs.* amoeboid) of IBA1^+^ cells was counted across the superior and inferior retina using two retinal sections per mouse. GFAP expression was analyzed by fluorescence intensity from the inner limiting membrane to the outer limiting membrane, as well as GFAP extension length using images captured with the A1^+^ confocal microscope at 20x magnification. Using ImageJ V2.0 software, intensity analysis was performed 0.5mm superior to the optic nerve and calculated as a relative intensity from controls.

#### TUNEL assay

Terminal deoxynucleotidyl transferase (Tdt) dUTP nick end labelling (TUNEL), was used as a measure of photoreceptor cell death. TUNEL *in situ* labelling was performed on retinal cryosections using a Tdt enzyme (Cat# 3333566001, Sigma-Aldrich, MO, United States) and biotinylated deoxyuridine triphosphate (dUTP) (Cat# 11093070910, Sigma-Aldrich, MO, United States) as previously described (27). Images of TUNEL staining were captured with the A1^+^Nikon confocal microscope at 20 and 40x magnification. The total number of TUNEL^+^ cells were counted including both the superior and inferior retina using two retinal sections per animal.

To further quantify photoreceptor survival, the thickness of the ONL on retinal cryosections was determined by counting the number of nuclei rows (photoreceptor cell bodies) in the area of retinal lesion development (1 mm superior to the optic nerve head). Photoreceptor cell row quantification was performed five times per retina using two retinal cryosections at comparable locations per mouse.

#### *In situ* hybridization

To visualize the expression and localization of miRNA miR-155, *in situ* hybridization was performed on retinal cryosections using RNAscope^®^ 2.5 HD Assay-RED kit as per the manufacturer’s instructions. Deviations from the manufacturer’s instructions are described here. Post-fixation was conducted with 10% NBF as per the manufacturer’s instructions. For pre-treatment, sections were covered with RNAscope^®^ hydrogen peroxide for 10 minutes. Target retrieval was performed manually with RNAscope^®^ 1x Target Retrieval Reagent heated over a heat plate to a mild boil before stabilizing the temperature at between 98–102°C. Upon temperature stabilization, slides were fully submerged for 15 minutes exactly before being immediately submerged into distilled water. Slide pre-treatment with RNAscope^®^ Protease III was performed for 15 minutes in a humidity chamber at 40°C. Fast Red reagent was used for signal detection for up to 10 minutes at RT. Abundant miRNAs such as U6 and miR-124 only required 30 second exposure to the Fast Red reagent for full development before the reaction was stopped. Counterstaining was not performed. Slides were baked at 60°C and coverslips added with Aqua-Poly-Mount (Polysciences Inc, PA USA) before storage at 4°C.

Localization of miR-124-3p within the retina was determined by *in situ* hybridization. A double DIG-labelled miR-124-3p miRCURY LNA miRNA Detection Probe (Exiqon, Vedbaek, Denmark) was used on retinal cryosections, which were hybridized for 1 h at 53°C as previously described (Chu-Tan et al., 2018). The bound probe was visualized using 5-bromo-4-chloro-3 indoyl phosphate (NBT/BCIP; Sigma-Aldrich Corp., St. Louis, MO, United States).

Bright field images were captured on the A1^+^ Nikon confocal microscope fitted with a DS-Ri1-U3 color camera at 20x magnification. All images were centered at the site of lesion located approximately 0.5mm superior to the optic nerve head.

### Gene expression analyses

#### RNA extraction

RNA extraction was performed using miRVana miRNA Isolation Kit (Thermo Fisher Scientific, MA, United States) according to the manufacturer’s instructions. The concentration and purity of each RNA sample was assessed using the ND-1000 spectrophotometer (Nanodrop Technologies, DE, United States).

#### cDNA synthesis from mRNA or miRNA templates

Following purification of RNA, cDNA was synthesized from 1 μg RNA using either the Tetro cDNA Synthesis Kit (Bioline Reagents, London, United Kingdom) from an mRNA template, or using the TaqMan MicroRNA RT kit (Thermo Fisher Scientific) from a miRNA template, according to manufacturers’ instructions.

#### qPCR analysis

The expression of inflammatory (*Casp1, Ccl2, Il-1β* and *Il-6*) and glial stress (*Gfap*) genes known to be involved in AMD pathogenesis was measured in PD and DR retinal lysates by qRT-PCR. The expression of miRNA miR-124-3p, and miR-155-5p was also investigated in retinal lysates across photo-oxidative damage and compared to DR controls. The expression of these genes and miRNA was measured using mouse specific TaqMan hydrolysis probes (**Table 1**) and TaqMan Gene Expression Master Mix (Thermo Fisher Scientific, MA, United States). Reactions were performed in technical duplicates in a 384-well format using a QuantStudio 12 K Flex RT-PCR machine (Thermo Fisher Scientific, MA, United States). Data was analyzed using the comparative C_t_ method (ΔΔC_t_) and results are presented as percent change relative to control. Expression was normalized to reference gene glyceraldehyde-3-phosphate dehydrogenase (*Gapdh*) for mRNA, and small nuclear RNA *U6* for miRNA.

**Table 1:** Gene Expression TaqMan Probes (ThermoFisher Scientific).

Gene Symbols	Gene name	Catalogue number
***miR-124a***	Mmu-miR-124-3p	001182
***miR-155-5p***	Mmu-miR-155-5p	002571 (assay ID)
***U6 snRNA***	Small nuclear RNA U6	001973 (assay ID)
***Gapdh***	Glyceraldehyde-3-phosphatase dehydrogenase	Mm99999915_g1
***Casp1***	Caspase-1	Mm00438023_m1
***Ccl2***	Chemokine (C-C motif) ligand 2	Mm99999056_m1
***Il-1β***	Interleukin -1β	Mm00434228_m1
***Il-6***	Interleukin-6	Mm00446190_m1

#### High-throughput RNA sequencing and bioinformatics

RNA was dried in RNA stabilization tubes overnight (Azenta Life Sciences, Suzhou, China) and shipped to Azenta Life Sciences (Suzhou, China) for bulk RNA sequencing. Sequencing libraries were constructed with the Illumina TruSeq unstranded RNA library preparation kit using polyA selection for mRNA enrichment, then sequenced on the Illumina NovaSeq6000 platform acquiring ~20 million, 150 base-pair, paired-end reads per sample. Read phred scores, adapter/index contamination were checked with FastQC (Babraham Bioinformatics), then aligned to mouse genome (mm39) using HISAT2 aligner with default parameters. Alignments were summarized with featureCounts, genes with low expression (<1 count per million) were filtered out, and normalization factors for remaining genes were calculated using trimmed means of m (TMM) (28) method. Normalized counts were prepared for linear modelling using voom transformation (29) and then a statistical model was fitted using lmFit (30) and moderated t-statistics were computed using ebayes function. Tables of fold change and p-value estimates were generated with topTable function and genes with p-value < 0.05 and absolute fold changes>0.5 were deemed differentially expressed. Similarity matrices used for hierarchical clustering were computed using Euclidean distance calculations, then sample agglomeration was performed using average linkage. Gene ontology analysis was performed using Enrichr. Sample clustering was assessed using principal component analysis and hierarchical clustering employing single linkage for sample agglomeration and Euclidean distance as measure of similarity (prcomp and hclust base R functions). Temporal patterns in gene expression were identified using the TCseq R package. This packages first identifies differentially expressed genes by performing pairwise comparisons between control (DR mice) and PD mice (3h, 6h or 24h of PD), then performs temporal pattern analysis by fuzzy clustering to identify gene expression trends. Functional annotation was carried out using gene set enrichment analysis with the M2 Molecular Signature Database as reference (31). Using publicly available single cell RNA sequencing data (32) a reference dataset was created containing the top 100 most highly expressed genes for each cell type. The enrichment of microRNA targets in each cell type was then tested using Enrichr. Single cell data was processed using Seurat (33) using the following steps: SCTransform() ➔ RunPCA() ➔ RunUMAP() ➔ FindNeighbours() ➔ FindClusters(). Retinal cell types were annotated using ScType R package (34). Enrichr algorithm (35) using miRTarBase (36) as reference was utilized to study the enrichment of miRNA targets. Specifically, differentially expressed genes (adjusted p-value < 0.05) were submitted to Enrichr (35) and assessed for their overlap with miRNA targets reported in miRTarBase (36). P-values were then corrected for multiple comparison using Benjamini-Hochberg adjustment method and the resultant p-values were used to score the enrichment of miRNA targets.

### Statistical analyses

Statistical analysis for RNA sequencing was performed as specified above. Graphing was generated using ggplot2 package and Prism V7.0. Unpaired Student’s *t*-tests or one-way analysis of variance (ANOVA) were performed using Prism V7.0. p-values < 0.05 were deemed statistically significant. All data was expressed as the mean ± SEM.

### Data availability

Raw RNA sequencing files are accessible from the Sequencing Read Archive under project PRJNA934406, and are supplied in **Supplementary Tables 1**-**3**.

## Results

### Significant photoreceptor cell death is observed within 24 hours of photo-oxidative damage

**Figure 1:**
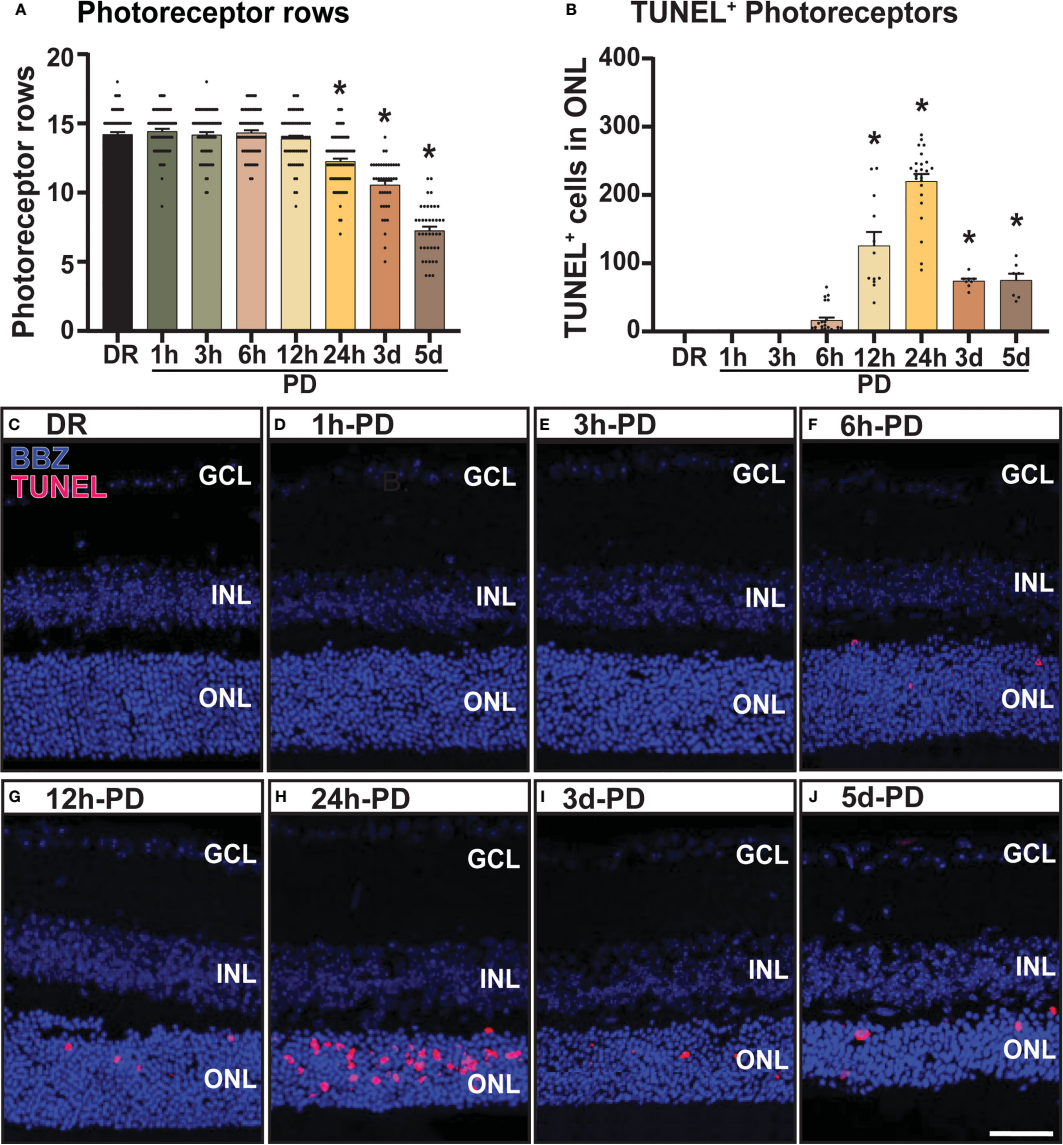
Photoreceptor cell death and outer nuclear layer thinning within 24 hours of photo-oxidative damage induced retinal degeneration. **(A)** Photoreceptor row counts on retinal cryosections of mice exposed to 1h-, 3h-, 6h-, 12h- and 24h-PD, showed a significant and progressive loss of photoreceptor rows from 24h-PD, compared to DR controls. **(B)** TUNEL^+^ cells (red) in the ONL were significantly increased compared to DR controls from 12h-PD to 5d-PD, with peak number of TUNEL^+^ cells at 24h-PD. **(C–J)** Representative confocal images depict progressive ONL thinning between **(G)** 12h-PD and **(J)** 5d-PD, and increased TUNEL^+^ cells in the ONL from **(F)** 6h-PD, peaking at **(H)** 24h-PD. *Significance using a one-way ANOVA, p < 0.05 and error bars indicate SEM. Scale bar = 50 μM. n = 12. Ganglion Cell Layer (GCL), Inner Nuclear Layer (INL), Outer Nuclear Layer (ONL).

Long periods (up to 5-7 days) of photo-oxidative damage (PD) have been well-established in wild-type C57BL/6J with no underlying genetic mutations, to induce key pathogenic features of late-stage retinal degenerations such as AMD, including focal photoreceptor and RPE cell death, microglia/macrophage infiltration, and inflammatory cascades (16, 18, 37–39). However, to date this model has not yet been used to characterize early pathological features of degeneration, in particular those occurring prior to observable levels of photoreceptor cell death. Therefore, to investigate early pathogenic features of retinal degeneration, a time-course of “early” (prior to photoreceptor cell death) and “late” degeneration was determined by subjecting mice to PD for short (<24h) and long periods of time (3-5 days). Levels of photoreceptor cell death were measured using photoreceptor row counts and a TUNEL assay. Compared to dim-reared (DR) controls, no significant photoreceptor row loss was observed between 1h-PD and 12h-PD (**Figure 1A**, *p* > 0.05), however a significant and progressive reduction in photoreceptor rows was observed at 24h-PD, 3d-PD and 5d-PD (**Figure 1A**, *p* < 0.05). To verify photoreceptor cell death, TUNEL^+^ cells in the ONL were counted and compared to DR controls. A significant increase in the number of TUNEL^+^ cells were observed in the ONL from 12h-PD to 5d-PD, peaking at 24h-PD (**Figure 1B**, *p* < 0.05), consistent with the observed ONL thinning shown at 24h-PD in **Figure 1A**. Representative confocal images (**Figures 1C–J**) depict photoreceptor row thinning from 24h-PD, with a small but significant increase in the number of TUNEL^+^ (red) cells shown in the ONL from 6h-PD, and higher levels particularly apparent by 24h-PD. Collectively these results indicate that, using PD, “early” degeneration can be defined up to 6h-PD, with “late degeneration” at 24h-PD shown by chronic and progressive ONL thinning and cell death.

### Early recruitment and activation of immune cells by 6h photo-oxidative damage-induced retinal degeneration precedes photoreceptor cell death

**Figure 2:**
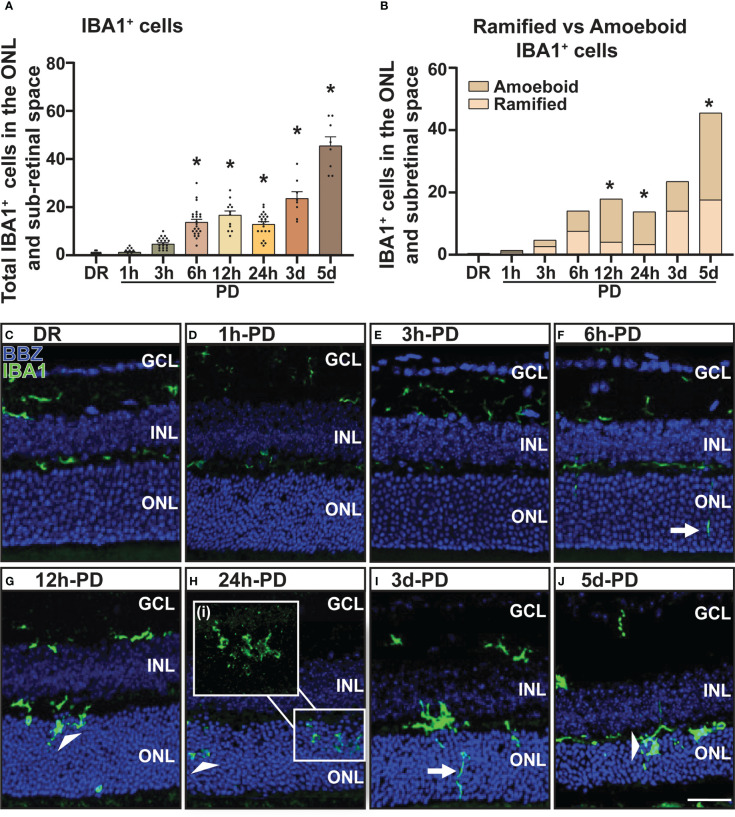
Increased presence and activation of IBA1^+^ microglia/macrophages in the outer retina from 6h-PD. IBA1 labelling was used to indicate microglia and macrophage recruitment to the outer retina (ONL and subretinal space). **(A)** IBA1^+^ cells were found to progressively increase in the outer retina from 6h-PD to 5d-PD compared to DR control. **(B)** In addition, a significantly greater number of amoeboid (activated; white arrow head) compared to ramified (resting; white arrows) IBA1^+^ cells was found at 12h-PD, 24h-PD and 5d-PD. **(C–J)** Representative confocal images show increased presence and activation of IBA1^+^ microglia/macrophages from 12h-PD to 5dPD compared to DR controls. *Significance using a one-way ANOVA, *p* < 0.05 and error bars indicate SEM. Scale bar = 50 μM. *n* = 12. Ganglion Cell Layer (GCL), Inner Nuclear Layer (INL), Outer Nuclear Layer (ONL).

The recruitment/activation of resident immune cells (microglia), and infiltration of peripheral immune cells (macrophage) into the outer retina is a characteristic feature of retinal degenerations and plays a key role in mediating retinal cell death (2, 40). Therefore, to further characterize the early pathological features of retinal degeneration, the presence and activation state of retinal immune cells (microglia/macrophages) was measured using IBA1 immunohistochemistry. From these results it was observed that compared to DR controls, the presence of IBA1^+^ cells (green) in the outer retina (ONL and subretinal space) was significantly increased as early as 6h-PD, and progressively increased throughout the damage paradigm (**Figure 2A**, *p* < 0.05). Further, it was also observed that from 12h-PD to 5d-PD, there was an increased morphological shift of IBA1^+^ cells from ramified (resting; white arrow) to amoeboid state (white arrow head), indicating an activated state (**Figure 2B**). Representative images show increased IBA1^+^ presence in the outer retina from 6h-PD, and a potentially more activated state with increased amoeboid compared to ramified IBA1^+^ cells (**Figures 2C–J**). Taken together these results demonstrate early immune cell recruitment and activation, which precedes significant levels of photoreceptor cell death observed from 12h-24h-PD.

### Progressive and early rise in retinal inflammatory gene expression during photo-oxidative damage-induced retinal degeneration

**Figure 3:**
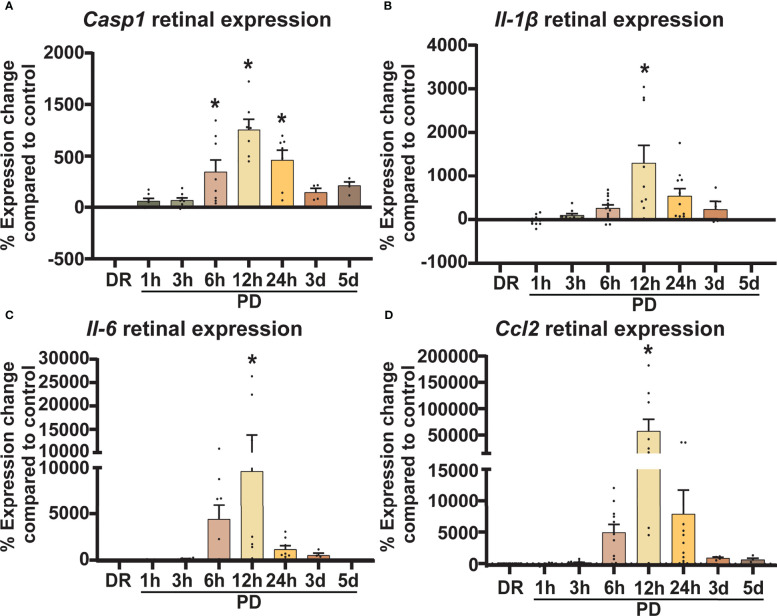
Retinal inflammatory gene expression shows a sharp early increase in response to photo-oxidative damage. Retinal expression of classic inflammatory genes known to be involved in AMD was measured using qRT-PCR. **(A–D)** Casp1, Il-1β, Il-6, and Ccl2 similarly show an increase in expression compared to DR controls from 6h-PD, with significant and peak expression at 12h-PD. The expression profile of all genes shows a gradual decrease from 24h-5d PD. *Significance using a one-way ANOVA, p < 0.05 and error bars indicate SEM. n = 12.

Inflammation is a hallmark pathological feature in the onset and progression of retinal degenerations including AMD, playing a key role in immune cell recruitment and retinal cell death (40). Therefore, to correlate levels of retinal inflammation to cell death and immune cell presence, gene expression of key inflammatory markers was measured in retinal lysates using qRT-PCR. The expression of inflammasome cleavage protease *Casp1*, was significantly increased between 6h-PD and 24h-PD (**Figure 3A**, *p* < 0.05), with downstream cleavage product pro-inflammatory cytokine interleukin-1β *(Il-1β)* also significantly increased at 12h-PD compared to DR controls (**Figure 3B**, *p* < 0.05). In addition, cytokine/chemokines *Il-6* and *Ccl2*, were also shown to be significantly upregulated at 12h-PD (**Figures 3C, D**, *p* < 0.05), consistent with the increased presence and activation of IBA1^+^ immune cells shown in **Figure 2**. These results suggest that early (6h-PD) molecular markers of degeneration can be observed prior to the onset of major photoreceptor cell death by 12-24h-PD and support the role of immune cell presence and inflammation as key drivers contributing to photoreceptor cell death.

### Retinal glial stress marker GFAP increases early and progressively across photo-oxidative damage-induced retinal degeneration

**Figure 4:**
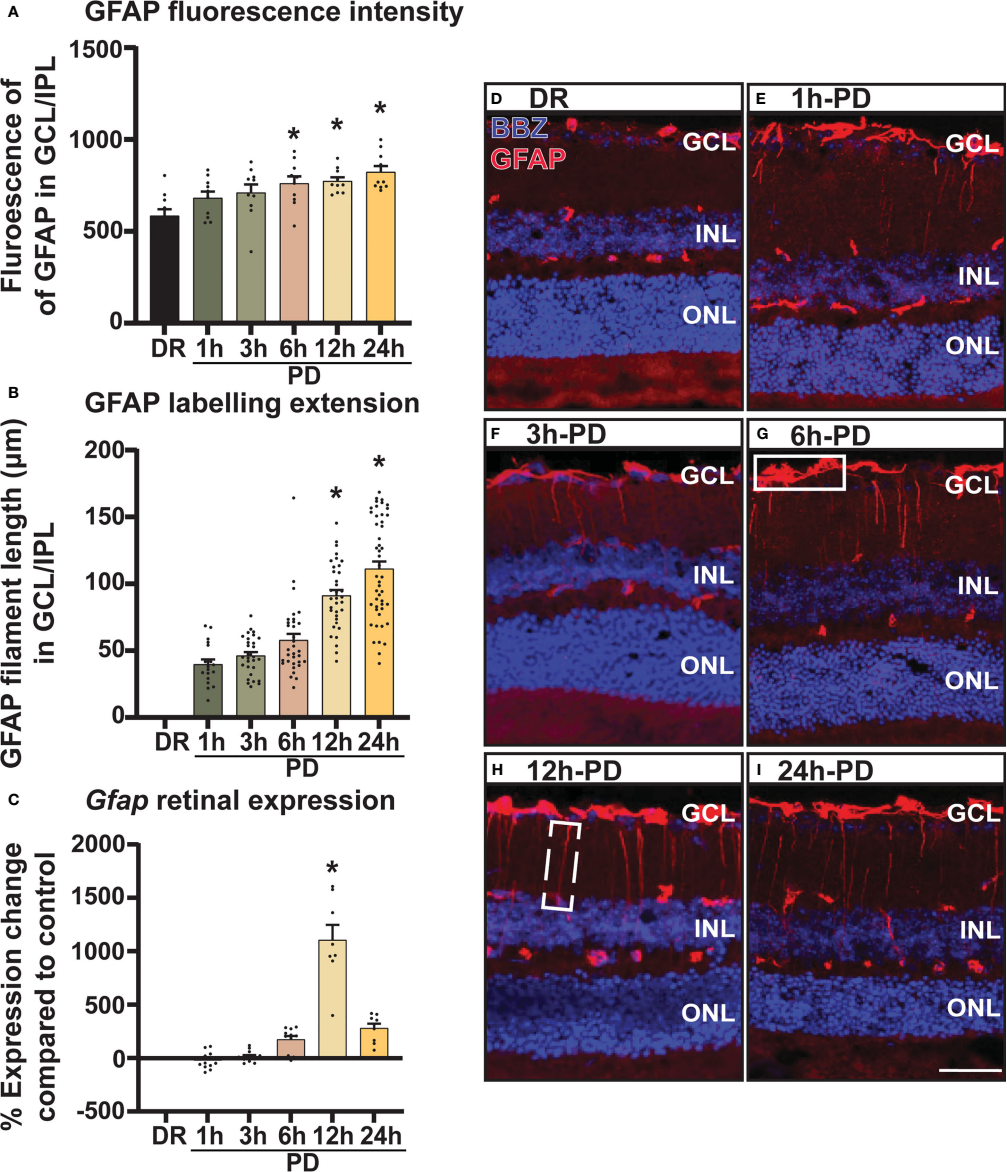
GFAP labelling demonstrates intensifying Müller cell stress from 6h-PD. GFAP labelling was used to indicate retinal Müller glia cell stress. **(A)** Fluorescence mean color intensity [white box; **(G)**] of GFAP in the GCL and IPL of the superior retina was found progressively increase across PD, with significant intensity compared to DR controls from 6h-PD to 24h-PD (*p* < 0.05, *n* = 4). In addition, **(B)** GFAP labelling length (dashed white box, H) also progressively increased across PD, and was significant at 12h-PD and 24h-PD compared to DR controls (*p* < 0.05, *n* = 4). **(C)** qRT-PCR shows increased *Gfap* expression in retinal lysates at 12h-PD (*p* < 0.05, *n* = 12). **(D–I)** Representative confocal images of the superior retina shows progressively increased GFAP labelling intensity and GFAP length in the GCL and IPL from 6h-PD to 24h-PD compared to DR controls. *Significance using a one-way ANOVA, *p* < 0.05 and error bars indicate SEM. Scale bar = 50 μM. *n* = 4-12.

In addition to inflammation, glial stress is a known pathological feature of retinal degenerations. Therefore, to investigate the onset and relative timing of glial stress in relation to cell death, the expression pattern of glial acidic fibrillary protein (GFAP), a marker of Müller glia cell stress was measured and quantified across PD. Results showed that both mean fluorescence intensity (**Figure 4A**) and GFAP labelling (**Figure 4B**) in the GCL and inner plexiform layer (IPL) increased progressively across PD, with a significant increase shown from 6h-PD for fluorescence intensity (*p* < 0.05) and 12h-PD for labelling length (*p* < 0.05). Further, gene expression analyses showed a significant increase in *Gfap* expression in retinal lysates at 12h-PD (**Figure 4C**, *p* < 0.05). Representative confocal images depict the increased GFAP (red) fluorescence (white box; G) and GFAP labelling length (dashed box; H) in the GCL/IPL at 6h-PD, 12h-PD and 24h-PD (**Figures 4D-I**). These results support that along with inflammation, early glial stress precedes and contributes to photoreceptor cell death. Further, these collective findings demonstrate the use of PD to incite early pathological features contributing to retinal degeneration, with both glial inflammation and stress observed and measured as early as 6h-PD.

### RNA sequencing identifies distinct transcriptomic profiles in the retina following short exposure to photo-oxidative damage

**Figure 5:**
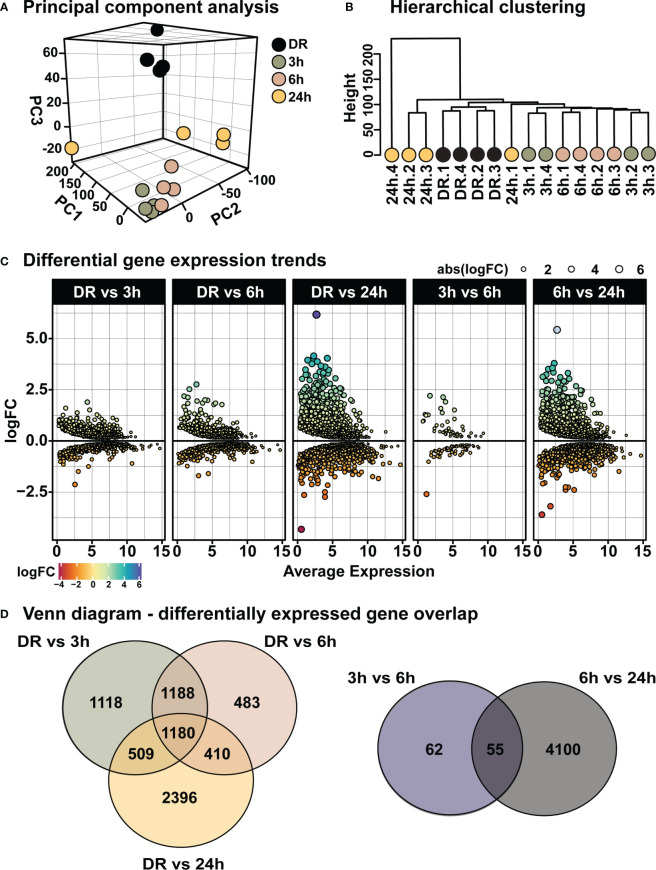
Distinct transcriptomic profiles in the retina following short exposure to photo-oxidative damage. **(A)** PCA shows between-group clustering, with some within-group variation shown at 24h-PD. These clustering profiles can also be shown by **(B)** hierarchical clustering. **(C)** Differential gene expression analysis shows distinct gene expression profiles at each time-point. **(D)** Venn diagrams show unique and shared differentially expressed genes at each time-point comparison. Differentially expressed genes determined by FDR < 0.05, FC > 1.5. *n* = 4.

From the above results a time-course of “early” retinal degeneration was determined by 6h-PD, with measurable levels of inflammation and glial stress observed prior to major photoreceptor cell loss shown by 24h-PD (“late” degeneration). Therefore, retinas from time periods of 3h-PD (no evidence of pathological markers of degeneration), 6h-PD (early detectable degenerative markers) and 24h-PD (photoreceptor row thinning and peak of cell death and late degeneration pathologies), along with DR (controls) were sent for RNA sequencing to identify transcriptomic changes controlling these pathogenic stages (**Supplementary Table 1**). Following normalization (**Supplementary Figures 1A-F**), principal component analysis (PCA) and hierarchical clustering identified distinct clustering between groups, with 3h-PD and 24h-PD samples showing some within-group variations (**Figures 5A, B**). Differential gene expression analysis (**Supplementary Figure 1G**) showed distinct gene expression profiles at each time-point comparison (**Figure 5C**; **Supplementary Table 2**, **Supplementary Figure 1G**; FDR < 0.05, FC > 1.5), with 1118, 483 and 2396 unique genes identified between DR and 3h-PD, 6h-PD and 24h-PD groups respectively, as well as 62 and 4100 unique genes between 3h-PD and 6h-PD, and 6h-PD and 24h-PD, as shown in Venn diagrams (**Figure 5D**). Taken together these results show distinct transcriptomic profiles at each time-point following short exposure to photo-oxidative damage, with significant gene expression changes found as early as 3h-PD.

### Early enrichment of inflammatory pathways contributes to progressive retinal degeneration

**Figure 6:**
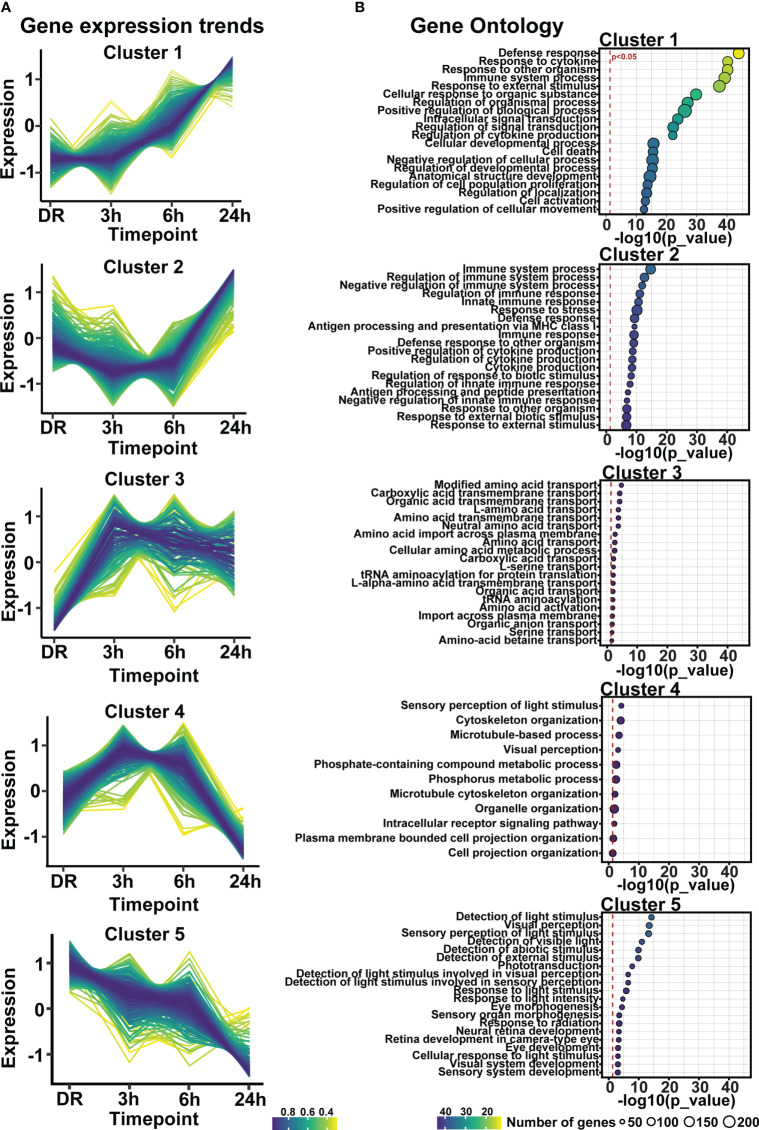
Gene expression profiles regulating inflammation, visual processing, metabolism, and amino-acid transport are altered in response to short exposure to photo-oxidative damage. **(A)** Fuzzy clustering analysis identified five trends of differentially expressed genes across 24h-PD. **(B)** Gene ontology analysis of genes within each cluster identified biological processes of inflammation, amino-acid transport, metabolism, and visual processing. **(A)** Color scheme indicates membership of gene to each cluster, **(B)** Significance p < 0.05, circle size in relation to number of genes associated with each GO term. n=4.

**Figure 7:**
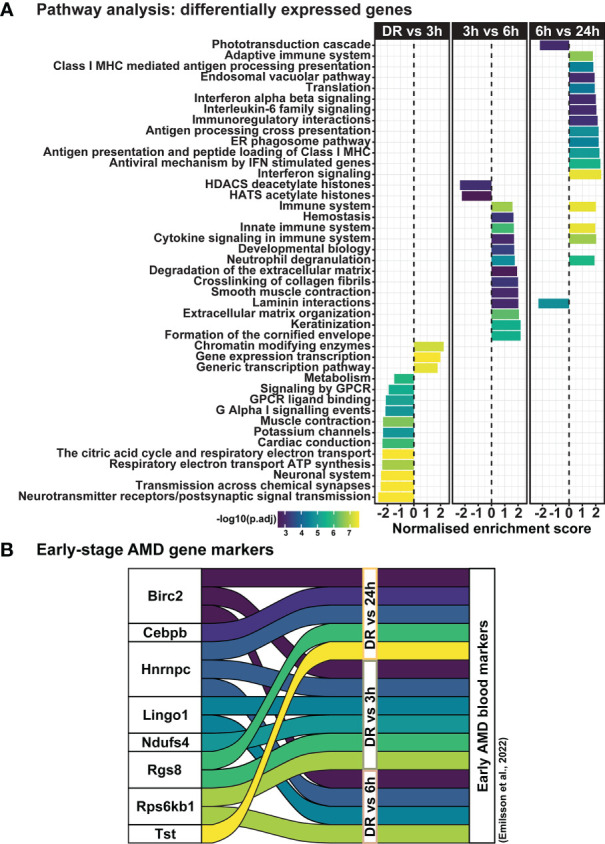
Dynamic shift in gene expression profile and pathway regulation across degeneration. **(A)** Pathway analysis shows unique biological processes occur across degeneration, with reduced enrichment of homeostatic metabolic and signal transmission processes between DR and 3h-PD groups, but increasing enrichment of inflammatory pathways from 3h-PD. Decreased enrichment of phototransduction cascade pathways can also be seen between 6h-PD to 24h-PD. **(B)** Comparison between upregulated early-stage AMD biomarkers in patient serum and in early time-course model of retinal degeneration shows overlapping markers. Significance *p < 0.05*, *n=4*.

Clustering analysis was performed on all differentially expressed genes relative to DR (controls), to identify gene expression trends associated with both early and late stages of photo-oxidative damage-induced degeneration. Results identified five main clusters with distinct gene expression trends (**Figure 6A**; **Supplementary Table 3**), with genes within cluster 1 increasing in expression progressively across photo-oxidative damage from 3h-PD to 24h-PD, while genes in cluster 5 decreased progressively from DR to 24h-PD. Genes within cluster 2 conversely showed a decreasing trend in expression from DR to 3-6h-PD before increasing in expression by 24h, while genes within clusters 3 and 4 both increased in expression from DR, peaking by 3-6h-PD before decreasing in expression by 24h-PD (**Figure 6A**). To identify the biological processes associated with genes within each cluster, gene ontology (GO) analysis was performed. Results showed that genes within each cluster were associated with terms relating to inflammation (clusters 1 and 2), amino acid transport (cluster 3), metabolism and cellular organization processes (cluster 4), and visual processing and perception (cluster 5) (**Figure 6B**). Pathway analysis of differentially expressed genes further highlighted that the most significantly enriched pathways were largely unique to each of the timepoint investigated, with decreased enrichment of metabolic and signal transmission pathways shown between DR to 3h-PD, increased enrichment of innate immune pathways between 3h-PD to 6h-PD and increased enrichment of adaptive and additional innate immune processes along with decreased enrichment of phototransduction processes found between 6h-PD to 24h-PD (**Figure 7A**). Collectively these results demonstrate that homeostatic biological processes are altered early in response to photo-oxidative damage <3h-PD, with increasing enrichment of inflammatory pathways as early between 3h-PD and 6h-PD likely contributing to progressive degeneration by 24h-PD. Finally, to validate this model of early retinal degeneration, identified upregulated protein serum markers from human patients with AMD (41) were cross referenced to genes across the early time-course model. Out of 15 potential early AMD markers identified in works by Emilsson et al, (2022) (41), 8 genes were also found within our early time-course dataset. Of these, 6 gene markers were expressed between DR and 3h-PD groups, 4 between DR and 6h-PD, and 5 between DR and 24h-PD groups (**Figure 7B**). Taken together these results highlight the validity of the use of short-exposure to photo-oxidative damage as a model of early degeneration and demonstrate the unique transcriptional changes that occur across degeneration to contribute to late-stage disease.

### Distinct inflammatory trends modulate early inflammation in response to photo-oxidative damage-induced degeneration

**Figure 8:**
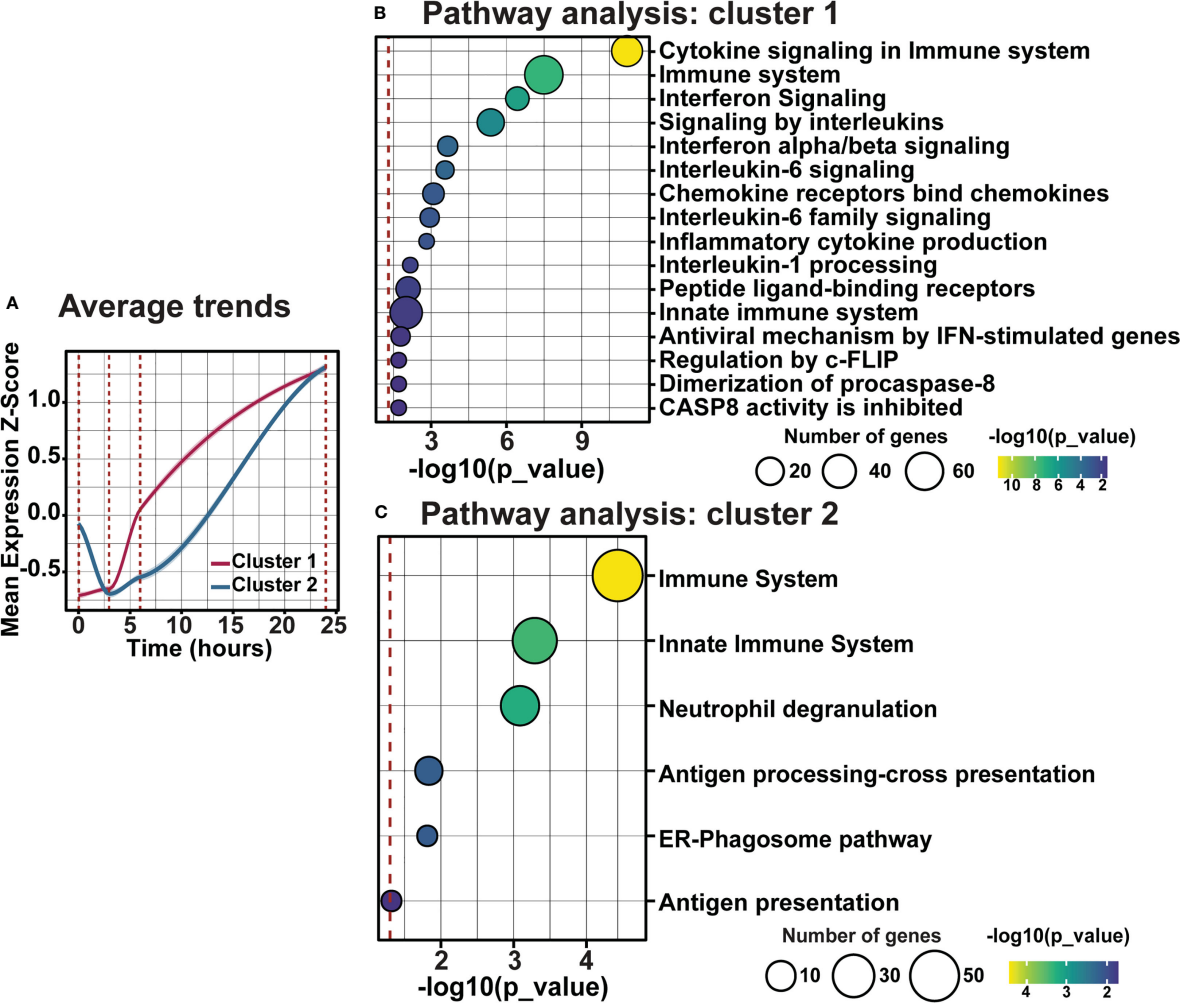
Distinct inflammatory trends modulate early inflammation in response to photo-oxidative damage-induced degeneration. **(A)** Average trend analysis shows two distinct gene clusters profiles regulating inflammation across photo-oxidative damage. **(B, C)** Pathway analyses of clusters 1 and 2 show two distinct inflammatory pathways in response to early photo-oxidative damage-induced degeneration. Significance *p < 0.05, n =4*.

As clustering, GO, and pathway analyses identified a key role for early inflammatory pathway enrichment in the progression of retinal degeneration, trend and pathway analyses were performed on genes within clusters 1 and 2. Examining the average fold change for genes in cluster 1 and 2 across the current photo-oxidative damage paradigm indicates that genes in cluster 1 are increasing progressively, while genes within cluster 2 decreased in expression from DR at 3h-PD before increasing in expression until 24h-PD (**Figure 8A**). Pathway analysis showed that genes within cluster 1 were associated with pathways regulating pro-inflammatory innate immune processes such as interleukin, chemokine and cytokine signaling (**Figure 8B**, *p < 0.05*), while genes within cluster 2 were associated with neutrophil degranulation along with antigen presentation and processing pathways, (**Figure 8C**, *p < 0.05*) suggesting a shift to adaptive immune processes may also be occurring. Finally, heat maps demonstrate distinct genes profiles of differentially expressed genes within clusters 1 and 2 (**Supplementary Figures 2A, B**). These results suggest that intervention prior to 3h-PD may prevent a shift to early degeneration onset, with a reduction in innate defense mechanisms leading to pathological inflammation.

### Key microRNA regulate early inflammation in photo-oxidative damage-induced retinal degeneration

**Figure 9:**
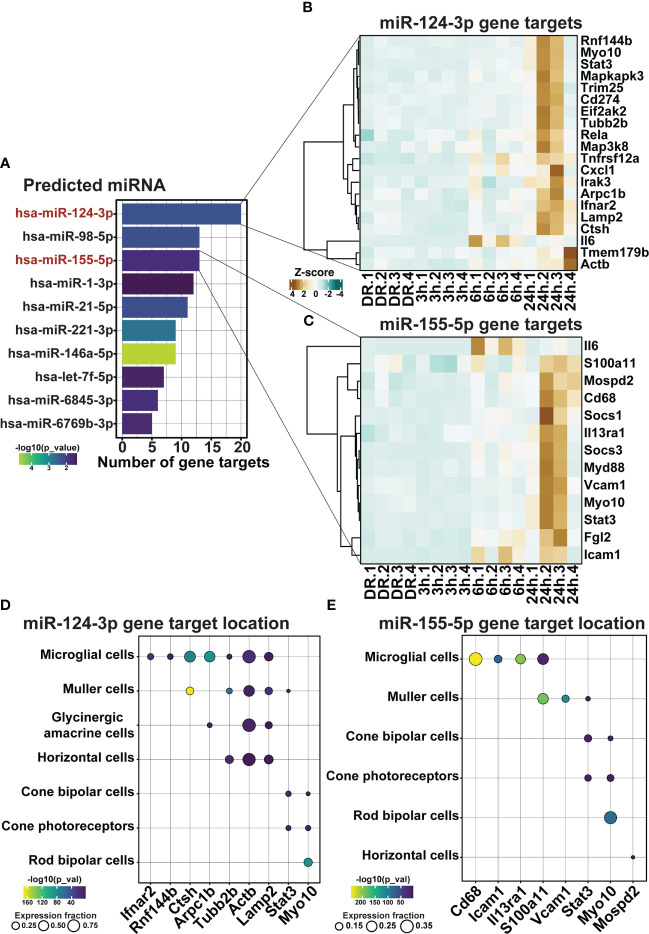
miR-155-5p and miR-124-3p associated with the regulation of retinal inflammation in early degeneration. **(A)** Top 10 significantly predicted miRNA which regulate inflammatory genes within clusters 1 and 2. **(B, C)** Predicted mRNA targets in dim-reared mice within clusters 1 and 2 of miR-124-3p and miR-155-5p**. (D, E)** miRNA-mRNA target cellular locations in the retina. Significance determined by *p < 0.05, n = 4*.

Finally, as the use of miRNA represents a strong potential therapeutic strategy for repressing inflammation due to their unique multi-target binding properties, miRNA-mRNA target analysis was performed on differentially expressed genes within clusters 1 and 2. Out of the top 10 significant miRNA, miR-124-3p and miR-155-5p, both known to play key roles in AMD progression (23–25, 42), were found to have the first and third highest number of predicted gene targets within clusters 1 and 2 (**Figure 9A**, *p < 0.05*), with 20 (**Figure 9B**) and 13 (**Figure 9C**) predicted targets each, respectively. To determine the potential cellular location of these predicted miRNA targets, the expression of these targets across retinal cell types was investigated using publicly available single cell RNA data from healthy (dim-reared) C57Bl/6J mice (**Supplementary Figure 1H**). Results identified that predicted targets of miR-124-3p (**Figure 9D**) and miR-155-5p (**Figure 9E**) were expressed in cells of the inner retina, including a strong expression fraction of many targets in microglial immune cells as well as Müller glia, and a smaller proportion of targets with known expression in cone photoreceptors, and horizontal and bipolar cells (**Figures 9D, E**, *p < 0.05*).

**Figure 10:**
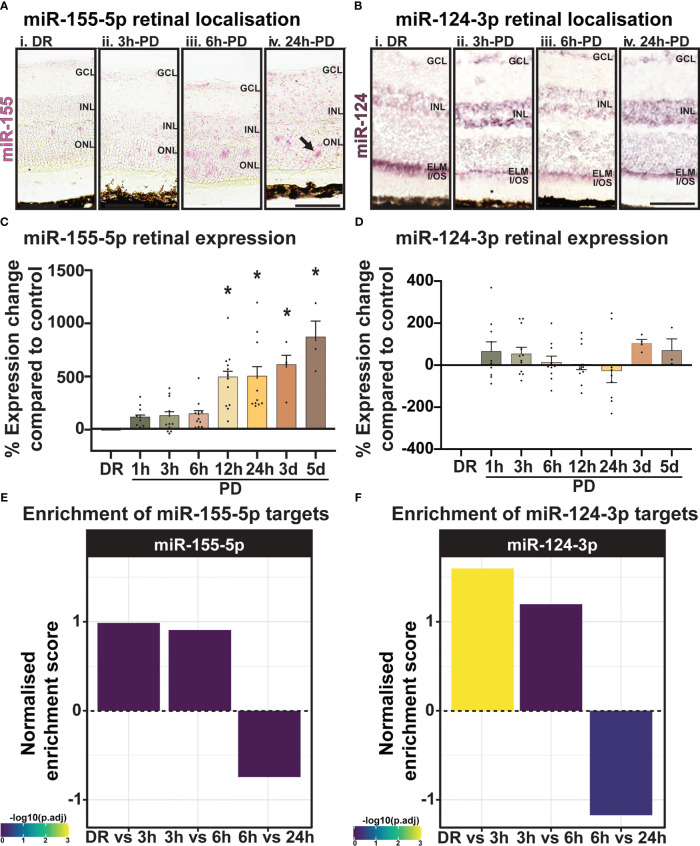
miR-155 expression progressively increases in the ONL across PD. **(A)** Using miRNAscope, the expression and localization of miR-155-5p (pink) was found to increase progressively during PD, with strong punctate labelling shown in the ONL at (A*iii*) 6h-PD and (A*iv*) 24h-PD compared to controls. Inner retinal (INL-GCL) labelling was also strongly observed at (*Aiv*) 24h-PD. **(B)** *In situ* hybridization for miR-124-3p (purple) shows (B*i*) strong labelling in the outer limiting membrane (OLM) in DR controls, with weak labelling in the INL and ONL. Labelling in the OLM decreased in appearance by (B*ii*) 3h-PD, with increased expression of miR-124-3p found in the INL. **(C, D)** qRT-PCR showed **(C)** progressively increased miR-155 expression levels in the retina in response to PD, with significant upregulation at each time point from 12h-PD to 5d-PD, **(D)** however no change in expression of miR-124-3p was seen at any time-point across degeneration. **(E, F)** Normalized enrichment of miR-155-5p and miR-124-3p targets across photo-oxidative damage show increased target enrichment between DR and 6h-PD, and decreased enrichment from 6h-PD to 24h-PD *Significance using a one-way ANOVA, *p < 0.05* and error bars indicate SEM. Scale bar = 50 μM. *n = 4.*

To validate the predicted miRNA target locations, the expression and localization of miR-155-5p (pink) and miR-124-3p (purple) was investigated using miRNAScope (**Figure 10A**) and *in-situ* hybridization (**Figure 10B**). While low-level miR-155-5p expression was seen in DR controls (**Figure 10Ai**) and at 3h-PD (**Figure 10Aii**) compared to negative controls, strong punctate miR-155 labelling was detected in the ONL and INL by 6h-PD (**Figure 10Aiii**). Further this labelling pattern appeared darker and more widespread through the ONL, INL, IPL and GCL by 24h-PD (**Figure 10Aiv**; black arrow). In comparison, strong miR-124-3p labelling was present in the outer limiting membrane (OLM) in DR controls, with weaker labelling in the ONL and INL, however by 3h-PD labelling in the OLM appeared to decrease while INL labelling was stronger in appearance (**Figures 10Bi-iv**). In support of predicted target locations, both miR-155-5p and miR-124-3p were found to be expressed in photoreceptors and cells of the inner retina.

Levels of miRNA were further quantified by qRT-PCR across the early and late photo-oxidative damage with miR-155-5p expression found to significantly increase in expression between 12h-PD to 5d-PD compared to DR controls (**Figure 10C**, *p *< 0.05). No change was found in the expression of miR-124-3p across any time point (**Figure 10D**, *p > 0.05*). Finally, to determine the overall potential strength of miR-124-3p and miR-155-5p interactions across photo-oxidative damage, miRNA target enrichment was calculated for both miR-124-3p and miR-155-5p across all differentially expressed genes at all time points between DR and 24h-PD. Normalized enrichment scores show increased enrichment of target genes for both miRNA between DR and 3h-PD, as well as 3h-PD and 6h-PD, and decreased target enrichment between 6h-PD and 24h-PD (**Figures 10E, F**). Collectively these results suggest that both expression and localization may play key roles in miRNA regulation of inflammation during photo-oxidative damage-induced degeneration and that targeting miR-124-3p and/or miR-155-5p therapeutically prior to 6h-PD may prevent loss of homeostatic processes and large-scale pathological inflammatory activation to reduce the progression of retinal degeneration.

## Discussion

Rodent models of neuronal degeneration, including *via the* use of photo-oxidative damage, have been integral in our understanding of molecular mechanisms and therapeutic strategies in retinal degenerative diseases, such as AMD (16, 43, 44). However, most rodent models of retinal degeneration are inherently suited to study late-stage AMD (16, 18, 39, 43, 44), preventing comprehensive understanding of early molecular changes which may contribute to disease onset and progression. In this work we therefore established an early model of retinal degeneration using short exposure to photo-oxidative damage (<24h-PD). Key findings from this study have shown that significant molecular changes in the retina can be discerned as early as 3h-PD, with these molecular changes preceding observable levels of immune cell recruitment and photoreceptor cell death. Specifically, we identified that homeostatic processes involved in metabolism, signal transmission and visual perception were downregulated by 3h-PD, while both distinct pro-inflammatory responses were found to occur from 3h-PD; likely contributing to the recruitment and activation of microglia/macrophage inflammatory cells seen at 6h-PD, and major observable photoreceptor cell loss by 24h-PD. Finally, we identified two key early enriched miRNA that were associated with the regulation of these inflammatory processes, miR-124-3p and miR-155-5p and visualized their dynamic and rapid movement throughout the retina in response to early degeneration. Taken together these findings show that short exposure to photo-oxidative damage can be used as a model of early retinal degeneration and could be used to identify early pathological molecules for intervention/targeting strategies to slow the progression to late-stage degeneration.

AMD is a chronic and progressive disease of the retina, characterized by photoreceptor cell death in the central retina, or macula, resulting in permanent irreversible blindness (2). While risk factors for AMD have been well defined (1, 11, 45, 46), they are also multifactorial in nature, creating a level of complexity in both disease diagnosis and intervention/treatment strategies. Further complicating diagnostic accuracies, early pathological signs, including small yellow lipid-filled deposits under the retina called drusen, as well as pigmentary abnormalities, can only be used to indicate the potential risk of AMD development, as they can also be found within the healthy aging population (1, 6, 12, 47, 48). Therefore, for those at risk, currently the only intervention strategy to combat AMD progression is dietary supplementation of AREDS2 formulation multivitamins, however this remains only efficacious in slowing the progression from intermediate to late-stage AMD (49, 50). Therefore, in order to identify more robust early therapeutic options, it is necessary to uncover early molecular changes within the retina. By identifying early pathological changes in the retina, molecular markers of early degeneration may be detected in the peripheral circulation and serve as diagnostic markers to compliment current clinical risk assessments (41, 51).

To date however, given the difficulties in identifying or classifying patients with early AMD, there is limited evidence of early retinal molecular changes, with only one recent publication identifying proteomic changes in the serum of early AMD patients (41). Using two different classification methods for early AMD (52, 53), Emilsson et al, (2022), identified in a large cohort study of 5457 patients, 15 proteins which had strong association with early-stage AMD including inflammatory regulators CEBPB, CCL1, CXCL17 and CFHR1. Further, in a 5 year follow up analysis of these patients, only one protein, Protein Arginine Methyltransferase 3 (PRMT3) was found to hold strong predictive value in the progression to geographic AMD (41). Results from our study, although measure retinal and not systemic changes, identified 8 of these 15 markers across our time-course of degeneration, with six early AMD markers found between DR and 6h-PD, one uniquely expressed between DR and 3h-PD groups, and two uniquely between DR and 24h-PD. Overall this finding supports the use of short exposure to photo-oxidative damage as a model for early retinal degenerations, and provides some validation to the markers identified by Emilsson et al. (2022). We propose that this model of early degeneration can be used to identify early molecular changes that may contribute to the progression from early, to intermediate and late-stage AMD, and help identify key time points for intervention therapies.

Inflammation is a central pathological contributor to the onset and progression of AMD (40), with the majority of existing therapeutic strategies aimed at targeting key inflammatory molecules and pathways known to play a role in disease pathogenesis (40, 54–58). In particular, therapeutics targeting cytokine, inflammasome and especially complement cascade components have dominated the pharmaceutical development landscape (58–60). While persistent low-grade inflammation or ‘parainflammation’ in the retina is required to maintaining retinal homeostasis (61, 62), as a consequence of accumulating cellular stress levels during normal aging, or for those genetically susceptible; chronic inflammation can build up causing tissue damage and ultimately vision loss (40, 62). Currently, the sequence of events which drives the retina from having seemingly protective, low-grade inflammation to a neurodegenerative, diseased state of chronic pathological inflammation is not well understood. Results from our early model of retinal degeneration can however be used to uncover early inflammatory changes, and assess their relation and functional significance to the onset of chronic inflammation and microglia/macrophage infiltration and activation. In this way, understanding the cause of glial activation and chronic inflammation may serve as a better marker for therapeutic intervention, and can aid in elucidating why infiltration has occurred and how it can be prevented.

The expression of known AMD inflammatory markers secreted from activated microglia/macrophages in retinal degenerations including chemokine *Ccl2*, inflammasome cleavage enzyme *Casp1*, and downstream pro-inflammatory cytokine *Il-1β* were detected in significant levels in the retina from 12h-PD to 24h-PD, in both qRT-PCR and RNA sequencing analyses, with notable inflammasome and complement cluster genes including Casp1, Casp8, C1qa, C3, C4b and C5ar1 peaking in expression at 24h-PD. However, these markers are more closely aligned with late-stage AMD pathology and are often reportedly detected in the serum, ocular fluids, retinal tissue and within drusen deposits of late-stage AMD patients (40, 51, 63–66), as well as in various animal models of AMD (22, 38, 67–69). While these inflammatory components are valuable diagnostic markers of late-stage disease, and may still represent potential therapeutic targets; given that the expression of these inflammatory markers coincides with microglia/macrophage activation and increased levels of photoreceptor cell death (22, 38, 67–69), their presence suggests that it may be too late for effective therapeutic strategies. We postulate that along with the multifaceted nature of AMD, targeting this late in disease progression and often using single-target drugs (58–60) is an ineffective strategy to combat this complex and debilitating disease, and is perhaps why to date, no candidate drugs have made it successfully through clinical trials (58–60). We therefore suggest that profiling early inflammatory gene and pathway changes which may be driving chronic inflammatory activation associated with late-stage AMD, can identify more viable therapeutic targets for early intervention strategies, and prevent the progression of this disease.

Significant molecular changes in the retina were identified in this work from 3h-PD, with both a downregulation of genes controlling homeostatic retinal processes including metabolism, signal transmission and visual perception, as well as significant upregulation of inflammatory pathways from 3h-PD. Specifically, we identified two distinct inflammatory clusters of genes that were regulated from 3h-PD, preceding any observable levels of activated microglia/macrophage infiltration into the retina or Müller cell gliosis, - significantly notable from 6h-PD. Out of these two inflammatory clusters of genes (cluster 1 and 2), unlike cluster 1 which increased progressively from control levels until 24h-PD, genes within cluster 2 were found to be downregulated between DR and 3h-PD, before increasingly in expression again past baseline levels from 6h-PD – corresponding to increased levels of microglia/macrophages in the outer retina. Further, genes within cluster 1 were more closely identified to control innate immune pathways including cytokine defense, regulation of cytokine production, and signaling by interleukins, while genes within cluster 2 were involved in both innate and adaptive immune responses, such as negative regulation of immune system processes, response to stress, neutrophil degranulation and antigen presentation. We suggest that collectively this data shows an initial inflammatory response to acute stress, however following sustained inflammation and overburdening of defense responses results in chronic upregulation of immune pathways which may be responsible for driving disease progression. This hypothesis can also be supported by results showing an initial downregulation of genes associated with pathways controlling homeostatic processes, with a significant reduction in phototransduction pathways apparent at 24h-PD coinciding with significantly upregulated cell death pathways and a notable loss of photoreceptor rows at this time point. These results strengthen the use of short-term photo-oxidative damage as an early model of AMD, and importantly demonstrate a sequence of molecular events that trigger the progression to late-stage degeneration. We hypothesize that therapeutic intervention prior to 6h-PD may prevent the cascading chronic inflammation and prevent the progression to late-stage AMD.

MicroRNA (miRNA) are considered master regulators of gene expression, as they are able to bind to, and repress multiple gene targets often within the same biological pathways, such as those involved in the innate immune response (70–72). This unique property makes them ideal diagnostic, therapeutic targets and drug discovery molecules for diseases such as AMD, where therapeutic regulation of over-active inflammatory pathways may preserve retinal homeostasis (21, 23, 73, 74). Results from our study identified key miRNA that were predicted to regulate inflammatory gene targets across clusters 1 and 2 in degeneration. Notably, miR-124-3p, and miR-155-5p, were the first and third most significantly associated miRNA, with both of these miRNA having strong diagnostic and therapeutic connections in retinal degenerations, including in AMD. We further noted in this work, that both miR-124-3p and miR-155-5p had rapid and distinct movement patterns across the retina during degeneration, with miR-124-3p translocation from the outer retina as previously described in Chu-Tan et al. (2018) to the inner retina (23) as early as 3h-PD. In contrast, miR-155-5p was shown to increase in expression in the inner retina, with punctate labelling seen in the inner nuclear layer (INL) from 6h-PD; coinciding with its demonstrated location in retinal immune cells (24), and increased expression in AMD (25). As both of these miRNA have known cellular locations within retinal glial cells, mRNA target analysis of genes within clusters 1 and 2 was performed using an online retinal single-cell sequencing dataset (32), identifying that a large number of their predicted miRNA targets were expressed in retinal cells within the inner retina, specifically with a large number of targets in retinal microglia and Müller glia. However, as this single-cell sequencing database was only collected from dim-reared healthy mice, target mRNA localization could not be performed on cluster 1 and 2 genes which are known to be upregulated during degeneration such as *Il-6*, *Myd88*, *Socs1, and Socs3;* but which are also known to be expressed by inner retinal glia in degeneration (26, 75–78). Finally, when evaluating all differentially expressed genes from this dataset, the total predicted binding targets for both miR-124-3p and miR-155-5p were found to decrease between 6h-PD and 24h-PD, suggesting that while the predicted inflammatory gene targets increased across degeneration, it is possible that homeostatic regulatory targets of these miRNA were decreased. This phenomenon supports work by Chu-Tan et al. (2021), that showed that in response to stress or disease, miRNA can change target binding partners. In fact, specifically in the retina miR-124-3p was shown to alter target binding, favoring inflammatory targets such as *Ccl2* following photo-oxidative damage-induced degeneration (42). Overall these results support that early intervention strategies including early targeting of these inflammatory regulator miRNA may allow for homeostatic regulation to continue, and prevent inflammatory cascades which ultimately result in photoreceptor cell death and permanent vision loss.

These results demonstrate that short exposure to photo-oxidative damage can be used to model the early changes in retinal inflammation, providing an insight into the early pathogenesis of AMD, and further allowing for the identification of key molecular changes in the retina which may contribute to late-stage disease progression. Inhibition of these early inflammatory molecules, or inflammatory pathway regulators such as miRNA, as well as detection in the systemic circulation represents ideal next steps for this work.

## Data availability statement

The data presented in the study are deposited in the Sequencing Read Archive NCBI repository, accession number PRJNA934406.

## Ethics statement

All experiments were conducted in accordance with the ARVO Statement for the Use of Animals in Ophthalmic and Vision Research and with approval from the Australian National University’s (ANU) Animal Experimentation Ethics Committee (AEEC) (Ethics ID: A2020/41; Rodent models and treatments for retinal degenerations).

## Author contributions

YW and RN: conceptualization; YW, AC, EW, and RN: methodology and data analysis. YW, AC, EW, and JCT: investigation; YW, AC, EW, RS, and RN: writing – draft, review, and editing; RN: supervision and funding acquisition. All authors contributed to the article and approved the submitted version.

## Supplementary material

Supplementary Figure 1 Box plots of **(A)** raw and **(B)** normalised read count distributions showing effective normalisation (TMM). Relative log expression (RLE) box plots for **(C)** raw and **(D)** normalised counts. RLE values for each gene were calculated by deriving the median expression of each gene across all samples then computing the deviation from this median in each sample. **(E)** Mean variance relationship of each gene following voom transformation. **(F)** Summary of sample level variation obtained using voomWithQualityWeights function. **(G)** Summary of number of differentially expressed genes with and without adjusted for sample specific weights. As sample specific variation was observed, and correcting for unequal variation had a significant effect on the number of differentially expressed genes, the corrections for unequal variation were incorporated in the final lmFit statistical model. **(H)** Uniform manifold approximation projection plot (UMAP) of cell types obtained from the reanalysis of retinal single cell RNA sequencing data (74).

Supplementary Figure 2 Distinct gene profiles within dual inflammatory pathways in response to early photo-oxidative damage. **(A, B)** Heat maps show the expression profile of differentially expressed genes within cluster 1 and 2, with unique genes within each cluster. Significance P<0.05, n=4.

## References

[1] Chakravarthy U Bailey CC Scanlon PH McKibbin M Khan RS Mahmood S. Progression from Early/Intermediate to advanced forms of age-related macular degeneration in a Large UK cohort: rates and risk factors. Ophthalmol Retina (2020) 4(7):662–72. doi: 10.1016/j.oret.2020.01.012 32144084

[2] Ambati J Atkinson JP Gelfand BD. Immunology of age-related macular degeneration. Nat Rev Immunol (2013) 13(6):438. doi: 10.1038/nri3459 23702979 PMC3941009

[3] Mitchell P. Eyes on the future: a clear outlook on age-related macular degeneration. Deloitte/Macular Degeneration Foundation Australia. (2011).

[4] Heath Jeffery RC Mukhtar SA Lopez D Preen DB McAllister IL Mackey DA. Incidence of newly registered blindness from age-related macular degeneration in Australia over a 21-year period: 1996–2016. Asia-Pacific J Ophthalmol (2021) 10(5):442–449. doi: 10.1097/APO.0000000000000415 34534144

[5] Ambati J Fowler BJ. Mechanisms of age-related macular degeneration. Neuron (2012) 75(1):26–39. doi: 10.1016/j.neuron.2012.06.018 22794258 PMC3404137

[6] García-Layana A Cabrera-López F García-Arumí J Arias-Barquet L Ruiz-Moreno JM. Early and intermediate age-related macular degeneration: update and clinical review. Clin Interv Aging (2017) 12:1579–87. doi: 10.2147/CIA.S142685 PMC5633280 29042759

[7] Hart KM Abbott C Ly A Kalff S Lek JJ Milston R. Optometry australia's chairside reference for the diagnosis and management of age-related macular degeneration. Clin Exp Optometry (2020) 103(3):254–64. doi: 10.1111/cxo.12964 31566818

[8] Schwartz R Loewenstein A. Early detection of age related macular degeneration: current status. Int J Retina Vitreous (2015) 1(1):20. doi: 10.1186/s40942-015-0022-7 27847613 PMC5088451

[9] Martinez B Peplow PV. MicroRNAs as diagnostic and prognostic biomarkers of age-related macular degeneration: advances and limitations. Neural Regener Res (2021) 16(3):440–7. doi: 10.4103/1673-5374.293131 PMC7996036 32985463

[10] Pucchio A Krance SH Pur DR Miranda RN Felfeli T. Artificial intelligence analysis of biofluid markers in age-related macular degeneration: a systematic review. Clin Ophthalmol (2022) 16:2463–76. doi: 10.2147/OPTH.S377262 PMC9369085 35968055

[11] Lambert NG ElShelmani H Singh MK Mansergh FC Wride MA Padilla M. Risk factors and biomarkers of age-related macular degeneration. Prog Retinal Eye Res (2016) 54:64–102. doi: 10.1016/j.preteyeres.2016.04.003 PMC4992630 27156982

[12] Ferris FL 3rd Wilkinson CP Bird A Chakravarthy U Chew E Csaky K. Clinical classification of age-related macular degeneration. Ophthalmology (2013) 120(4):844–51. doi: 10.1016/j.ophtha.2012.10.036 PMC11551519 23332590

[13] Bellezza I. Oxidative stress in age-related macular degeneration: Nrf2 as therapeutic target. Front Pharmacol (2018) 9:1280. doi: 10.3389/fphar.2018.01280 30455645 PMC6230566

[14] Hadziahmetovic M Malek G. Age-related macular degeneration revisited: from pathology and cellular stress to potential therapies. Front Cell Dev Biol (2021) 8. doi: 10.3389/fcell.2020.612812 PMC7868387 33569380

[15] Lau L-I Chiou S-H Liu CJ-L Yen M-Y Wei Y-H. The effect of photo-oxidative stress and inflammatory cytokine on complement factor h expression in retinal pigment epithelial cells. Invest Ophthalmol Visual Sci (2011) 52(9):6832–41. doi: 10.1167/iovs.11-7815 21743006

[16] Natoli R Jiao H Barnett NL Fernando N Valter K Provis JM. A model of progressive photo-oxidative degeneration and inflammation in the pigmented C57BL/6J mouse retina. Exp Eye Res (2016) 147:114–27. doi: 10.1016/j.exer.2016.04.015 27155143

[17] Zhao Z Sun T Jiang Y Wu L Cai X Sun X. Photooxidative damage in retinal pigment epithelial cells *via* GRP78 and the protective role of grape skin polyphenols. Food Chem Toxicol (2014) 74:216–24. doi: 10.1016/j.fct.2014.10.001 25447759

[18] Fletcher EL Jobling AI Greferath U Mills SA Waugh M Ho T. Studying age-related macular degeneration using animal models. Optometry Vision Sci (2014) 91(8):878. doi: 10.1097/OPX.0000000000000322 PMC4186726 24978866

[19] Krol J Busskamp V Markiewicz I Stadler MB Ribi S Richter J. Characterizing light-regulated retinal MicroRNAs reveals rapid turnover as a common property of neuronal MicroRNAs. Cell (2010) 141(4):618–31. doi: 10.1016/j.cell.2010.03.039 20478254

[20] Rutar M Natoli R Kozulin P Valter K Gatenby P Provis JM. Analysis of complement expression in light-induced retinal degeneration: synthesis and deposition of C3 by Microglia/Macrophages is associated with focal photoreceptor degeneration. Invest Ophthalmol Visual Sci (2011) 52(8):5347–58. doi: 10.1167/iovs.10-7119 21571681

[21] Zhu Q Sun W Okano K Chen Y Zhang N Maeda T. Sponge transgenic mouse model reveals important roles for the microRNA-183 (miR-183)/96/182 cluster in postmitotic photoreceptors of the retina. J Biol Chem (2011) 286(36):31749–60. doi: 10.1074/jbc.M111.259028 PMC3173082 21768104

[22] Wooff Y Man SM Aggio-Bruce R Natoli R Fernando N. IL-1 family members mediate cell death, inflammation and angiogenesis in retinal degenerative diseases. Front Immunol (2019) 10:1618. doi: 10.3389/fimmu.2019.01618 31379825 PMC6646526

[23] Chu-Tan JA Rutar M Saxena K Aggio-Bruce R Essex RW Valter K. MicroRNA-124 dysregulation is associated with retinal inflammation and photoreceptor death in the degenerating retina. Invest Ophthalmol Visual Sci (2018) 59(10):4094–105. doi: 10.1167/iovs.18-24623 PMC11647551 30098196

[24] Aggio-Bruce R Chu-Tan JA Wooff Y Cioanca AV Schumann U Natoli R. Inhibition of microRNA-155 protects retinal function through attenuation of inflammation in retinal degeneration. Mol Neurobiol (2020). 58(2):835–854 doi: 10.1007/s12035-020-02158-z PMC7843561 33037565

[25] Romano GL Platania CB Drago F Salomone S Ragusa M Barbagallo C. Retinal and circulating miRNAs in age-related macular degeneration: an *in vivo* animal and human study. Front Pharmacol (2017) 8:168. doi: 10.3389/fphar.2017.00168 28424619 PMC5371655

[26] Rutar M Natoli R Chia RX Valter K Provis JM. Chemokine-mediated inflammation in the degenerating retina is coordinated by müller cells, activated microglia, and retinal pigment epithelium. J Neuroinflammation (2015) 12(1):8. doi: 10.1186/s12974-014-0224-1 25595590 PMC4308937

[27] Natoli R Zhu Y Valter K Bisti S Eells J Stone J. Gene and noncoding RNA regulation underlying photoreceptor protection: microarray study of dietary antioxidant saffron and photobiomodulation in rat retina. Mol Vis (2010) 16:1801–1822. PMC2932490 20844572

[28] Robinson MD Oshlack A. A scaling normalization method for differential expression analysis of RNA-seq data. Genome Biol (2010) 11(3):R25. doi: 10.1186/gb-2010-11-3-r25 20196867 PMC2864565

[29] Law CW Chen Y Shi W Smyth GK. Voom: precision weights unlock linear model analysis tools for RNA-seq read counts. Genome Biol (2014) 15(2):R29. doi: 10.1186/gb-2014-15-2-r29 24485249 PMC4053721

[30] Ritchie ME Phipson B Wu D Hu Y Law CW Shi W. Limma powers differential expression analyses for RNA-sequencing and microarray studies. Nucleic Acids Res (2015) 43(7):e47. doi: 10.1093/nar/gkv007 25605792 PMC4402510

[31] Subramanian A Tamayo P Mootha Vamsi K Mukherjee S Ebert Benjamin L Gillette Michael A. Gene set enrichment analysis: a knowledge-based approach for interpreting genome-wide expression profiles. Proc Natl Acad Sci (2005) 102(43):15545–50. doi: 10.1073/pnas.0506580102 PMC1239896 16199517

[32] Fadl BR Brodie SA Malasky M Boland JF Kelly MC Kelley MW. An optimized protocol for retina single-cell RNA sequencing. Mol Vis (2020) 26:705–17. PMC7553720 33088174

[33] Hao Y Hao S Andersen-Nissen E Mauck WM Zheng S Butler A. Integrated analysis of multimodal single-cell data. Cell (2021) 184(13):3573–87.e29. doi: 10.1016/j.cell.2021.04.048 34062119 PMC8238499

[34] Ianevski A Giri AK Aittokallio T. Fully-automated and ultra-fast cell-type identification using specific marker combinations from single-cell transcriptomic data. Nat Commun (2022) 13(1):1246. doi: 10.1038/s41467-022-28803-w 35273156 PMC8913782

[35] Chen EY Tan CM Kou Y Duan Q Wang Z Meirelles GV. Enrichr: interactive and collaborative HTML5 gene list enrichment analysis tool. BMC Bioinf (2013) 14:128. doi: 10.1186/1471-2105-14-128 PMC3637064 23586463

[36] Huang HY Lin YC Li J Huang KY Shrestha S Hong HC. miRTarBase 2020: updates to the experimentally validated microRNA-target interaction database. Nucleic Acids Res (2020) 48(D1):D148–d54. doi: 10.1093/nar/gkz896 PMC7145596 31647101

[37] Jiao H Rutar M Fernando N Yednock T Sankaranarayanan S Aggio-Bruce R. Subretinal macrophages produce classical complement activator C1q leading to the progression of focal retinal degeneration. Mol neurodegeneration (2018) 13(1):45. doi: 10.1186/s13024-018-0278-0 PMC6102844 30126455

[38] Natoli R Fernando N Jiao H Racic T Madigan M Barnett NL. Retinal macrophages synthesize C3 and activate complement in AMD and in models of focal retinal degeneration. Invest Ophthalmol Visual sci (2017) 58(7):2977–90. doi: 10.1167/iovs.17-21672 28605809

[39] Pennesi ME Neuringer M Courtney RJ. Animal models of age related macular degeneration. Mol aspects Med (2012) 33(4):487–509. doi: 10.1016/j.mam.2012.06.003 22705444 PMC3770531

[40] Kauppinen A Paterno JJ Blasiak J Salminen A Kaarniranta K. Inflammation and its role in age-related macular degeneration. Cell Mol Life Sci CMLS (2016) 73(9):1765–86. doi: 10.1007/s00018-016-2147-8 PMC4819943 26852158

[41] Emilsson V Gudmundsson EF Jonmundsson T Jonsson BG Twarog M Gudmundsdottir V. A proteogenomic signature of age-related macular degeneration in blood. Nat Commun (2022) 13(1):3401. doi: 10.1038/s41467-022-31085-x 35697682 PMC9192739

[42] Chu-Tan JA Cioanca AV Feng Z-P Wooff Y Schumann U Aggio-Bruce R. Functional microRNA targetome undergoes degeneration-induced shift in the retina. Mol Neurodegeneration (2021) 16(1):60. doi: 10.1186/s13024-021-00478-9 PMC8406976 34465369

[43] Marc RE Jones B Watt C Vazquez-Chona F Vaughan D Organisciak D. Extreme retinal remodeling triggered by light damage: implications for age related macular degeneration. Mol vision (2008) 14:782. PMC2375357 18483561

[44] Soundara Pandi SP Ratnayaka JA Lotery AJ Teeling JL. Progress in developing rodent models of age-related macular degeneration (AMD). Exp Eye Res (2021) 203:108404. doi: 10.1016/j.exer.2020.108404 33340497

[45] Chakravarthy U Wong TY Fletcher A Piault E Evans C Zlateva G. Clinical risk factors for age-related macular degeneration: a systematic review and meta-analysis. BMC ophthalmol (2010) 10(1):31. doi: 10.1186/1471-2415-10-31 21144031 PMC3009619

[46] Ross RJ Verma V Rosenberg KI Chan CC Tuo J. Genetic markers and biomarkers for age-related macular degeneration. Expert Rev Ophthalmol (2007) 2(3):443–57. doi: 10.1586/17469899.2.3.443 PMC2000850 17917691

[47] Khan KN Mahroo OA Khan RS Mohamed MD McKibbin M Bird A. Differentiating drusen: drusen and drusen-like appearances associated with ageing, age-related macular degeneration, inherited eye disease and other pathological processes. Prog retinal eye Res (2016) 53:70–106. doi: 10.1016/j.preteyeres.2016.04.008 27173377

[48] Flores R Carneiro Â Tenreiro S Seabra MC. Retinal progression biomarkers of early and intermediate age-related macular degeneration. Life (Basel) (2021) 12(1):36. doi: 10.3390/life12010036 35054429 PMC8779095

[49] Carneiro Â Andrade JP. Nutritional and lifestyle interventions for age-related macular degeneration: a review. Oxid Med Cell Longevity (2017) 2017:6469138. doi: 10.1155/2017/6469138 PMC5244028 28154734

[50] Camelo S Latil M Veillet S Dilda PJ Lafont R. Beyond AREDS formulations, what is next for intermediate age-related macular degeneration (iAMD) treatment? potential benefits of antioxidant and anti-inflammatory apocarotenoids as neuroprotectors. Oxid Med Cell Longevity (2020) 2020:4984927. doi: 10.1155/2020/4984927 PMC7803142 33520083

[51] Kersten E Paun CC Schellevis RL Hoyng CB Delcourt C Lengyel I. Systemic and ocular fluid compounds as potential biomarkers in age-related macular degeneration. Surv Ophthalmol (2018) 63(1):9–39. doi: 10.1016/j.survophthal.2017.05.003 28522341

[52] Holliday EG Smith AV Cornes BK Buitendijk GH Jensen RA Sim X. Insights into the genetic architecture of early stage age-related macular degeneration: a genome-wide association study meta-analysis. PLoS One (2013) 8(1):e53830. doi: 10.1371/journal.pone.0053830 23326517 PMC3543264

[53] Jonasson F Fisher DE Eiriksdottir G Sigurdsson S Klein R Launer LJ. Five-year incidence, progression, and risk factors for age-related macular degeneration: the age, gene/environment susceptibility study. Ophthalmology (2014) 121(9):1766–72. doi: 10.1016/j.ophtha.2014.03.013 PMC4145014 24768241

[54] Guo H Callaway JB Ting JPY. Inflammasomes: mechanism of action, role in disease, and therapeutics. Nat Med (2015) 21:677. doi: 10.1038/nm.3893 26121197 PMC4519035

[55] Marneros AG. NLRP3 inflammasome blockade inhibits VEGF-a-induced age-related macular degeneration. Cell Rep (2013) 4(5):945–58. doi: 10.1016/j.celrep.2013.08.002 PMC3821550 24012762

[56] Yerramothu P Vijay AK Willcox MDP. Inflammasomes, the eye and anti-inflammasome therapy. Eye (2017) 32:491. doi: 10.1038/eye.2017.241 29171506 PMC5848281

[57] Kyosseva SV. Targeting MAPK signaling in age-related macular degeneration. Ophthalmol Eye Dis (2016) 8:23–30. doi: 10.4137/OED.S32200 27385915 PMC4920203

[58] Park DH Connor KM Lambris JD. The challenges and promise of complement therapeutics for ocular diseases. Front Immunol (2019) 10:1007. doi: 10.3389/fimmu.2019.01007 31156618 PMC6529562

[59] Qin S Dong N Yang M Wang J Feng X Wang Y. Complement inhibitors in age-related macular degeneration: a potential therapeutic option. J Immunol Res (2021) 2021:9945725. doi: 10.1155/2021/9945725 34368372 PMC8346298

[60] Halawa OA Lin JB Miller JW Vavvas DG. A review of completed and ongoing complement inhibitor trials for geographic atrophy secondary to age-related macular degeneration. J Clin Med (2021) 10(12):2580. doi: 10.3390/jcm10122580 34208067 PMC8230644

[61] Chen M Forrester JV Xu H. Dysregulation in retinal para-inflammation and age-related retinal degeneration in CCL2 or CCR2 deficient mice. PLoS One (2011) 6(8):e22818. doi: 10.1371/journal.pone.0022818 21850237 PMC3151263

[62] Xu H Chen M Forrester JV. Para-inflammation in the aging retina. Prog retinal eye Res (2009) 28(5):348–68. doi: 10.1016/j.preteyeres.2009.06.001 19560552

[63] Bradley DT Zipfel PF Hughes AE. Complement in age-related macular degeneration: a focus on function. Eye (Lond). (2011) 25(6):683–93. doi: 10.1038/eye.2011.37 PMC3178140 21394116

[64] Celkova L Doyle SL Campbell M. NLRP3 inflammasome and pathobiology in AMD. J Clin Med (2015) 4(1):172–92. doi: 10.3390/jcm4010172 PMC4470247 26237026

[65] Crabb JW. The proteomics of drusen. Cold Spring Harb Perspect Med (2014) 4(7):a017194–a. doi: 10.1101/cshperspect.a017194 PMC4066642 24799364

[66] Newman AM Gallo NB Hancox LS Miller NJ Radeke CM Maloney MA. Systems-level analysis of age-related macular degeneration reveals global biomarkers and phenotype-specific functional networks. Genome Med (2012) 4(2):16–. doi: 10.1186/gm315 PMC3372225 22364233

[67] Wooff Y Fernando N Wong JH Dietrich C Aggio-Bruce R Chu-Tan JA. Caspase-1-dependent inflammasomes mediate photoreceptor cell death in photo-oxidative damage-induced retinal degeneration. Sci Rep (2020) 10(1):1–20. doi: 10.1038/s41598-020-58849-z 32041990 PMC7010818

[68] Natoli R Fernando N Madigan M Chu-Tan JA Valter K Provis J. Microglia-derived IL-1β promotes chemokine expression by müller cells and RPE in focal retinal degeneration. Mol neurodegeneration (2017) 12(1):31. doi: 10.1186/s13024-017-0175-y PMC5404662 28438165

[69] Rutar M Natoli R Valter K Provis JM. Early focal expression of the chemokine Ccl2 by müller cells during exposure to damage-inducing bright continuous light. Invest Ophthalmol Vis Sci (2011) 52(5):2379–2388. doi: 10.1167/iovs.10-6010 PMC3081230 21228381

[70] Chu Y-W Chang K-P Chen C-W Liang Y-T Soh ZT Hsieh LC. miRgo: integrating various off-the-shelf tools for identification of microRNA–target interactions by heterogeneous features and a novel evaluation indicator. Sci Rep (2020) 10(1):1466. doi: 10.1038/s41598-020-58336-5 32001758 PMC6992741

[71] Wu S Huang S Ding J Zhao Y Liang L Liu T. Multiple microRNAs modulate p21Cip1/Waf1 expression by directly targeting its 3' untranslated region. Oncogene (2010) 29(15):2302–8. doi: 10.1038/onc.2010.34 20190813

[72] Taganov KD Boldin MP Chang K-J Baltimore D. NF-kappaB-dependent induction of microRNA miR-146, an inhibitor targeted to signaling proteins of innate immune responses. Proc Natl Acad Sci U S A (2006) 103(33):12481–6. doi: 10.1073/pnas.0605298103 PMC1567904 16885212

[73] Pawlick JS Zuzic M Pasquini G Swiersy A Busskamp V. MiRNA regulatory functions in photoreceptors. Front Cell Dev Biol (2020) 8:620249. doi: 10.3389/fcell.2020.620249 33553155 PMC7858257

[74] Tahamtan A Teymoori-Rad M Nakstad B Salimi V. Anti-inflammatory MicroRNAs and their potential for inflammatory diseases treatment. Front Immunol (2018) 9(1377). doi: 10.3389/fimmu.2018.01377 PMC6026627 29988529

[75] Rashid K Akhtar-Schaefer I Langmann T. Microglia in retinal degeneration. Front Immunol (2019) 10:1975. doi: 10.3389/fimmu.2019.01975 31481963 PMC6710350

[76] Droho S Cuda CM Perlman H Lavine JA. Macrophage-derived interleukin-6 is necessary and sufficient for choroidal angiogenesis. Sci Rep (2021) 11(1):18084. doi: 10.1038/s41598-021-97522-x 34508129 PMC8433398

[77] Wang T Tsirukis D Cho S Sun Y. The roles of SOCS3 in myeloid cell-derived neovascular endothelium formation in a laser-induced choroidal neovascularization mouse model. Invest Ophthalmol Visual Sci (2021) 62(8):642.

[78] Syeda S Patel AK Lee T Hackam AS. Reduced photoreceptor death and improved retinal function during retinal degeneration in mice lacking innate immunity adaptor protein MyD88. Exp Neurol (2015) 267:1–12. doi: 10.1016/j.expneurol.2015.02.027 25725353 PMC4417011

