# Supplementary figures and images for: Short exposure to photo-oxidative damage triggers molecular signals indicative of early retinal degeneration
Source: Front Immunol. 2023 Apr 27;14:1088654. doi: 10.3389/fimmu.2023.1088654 (PMC10174249; doi:10.3389/fimmu.2023.1088654)

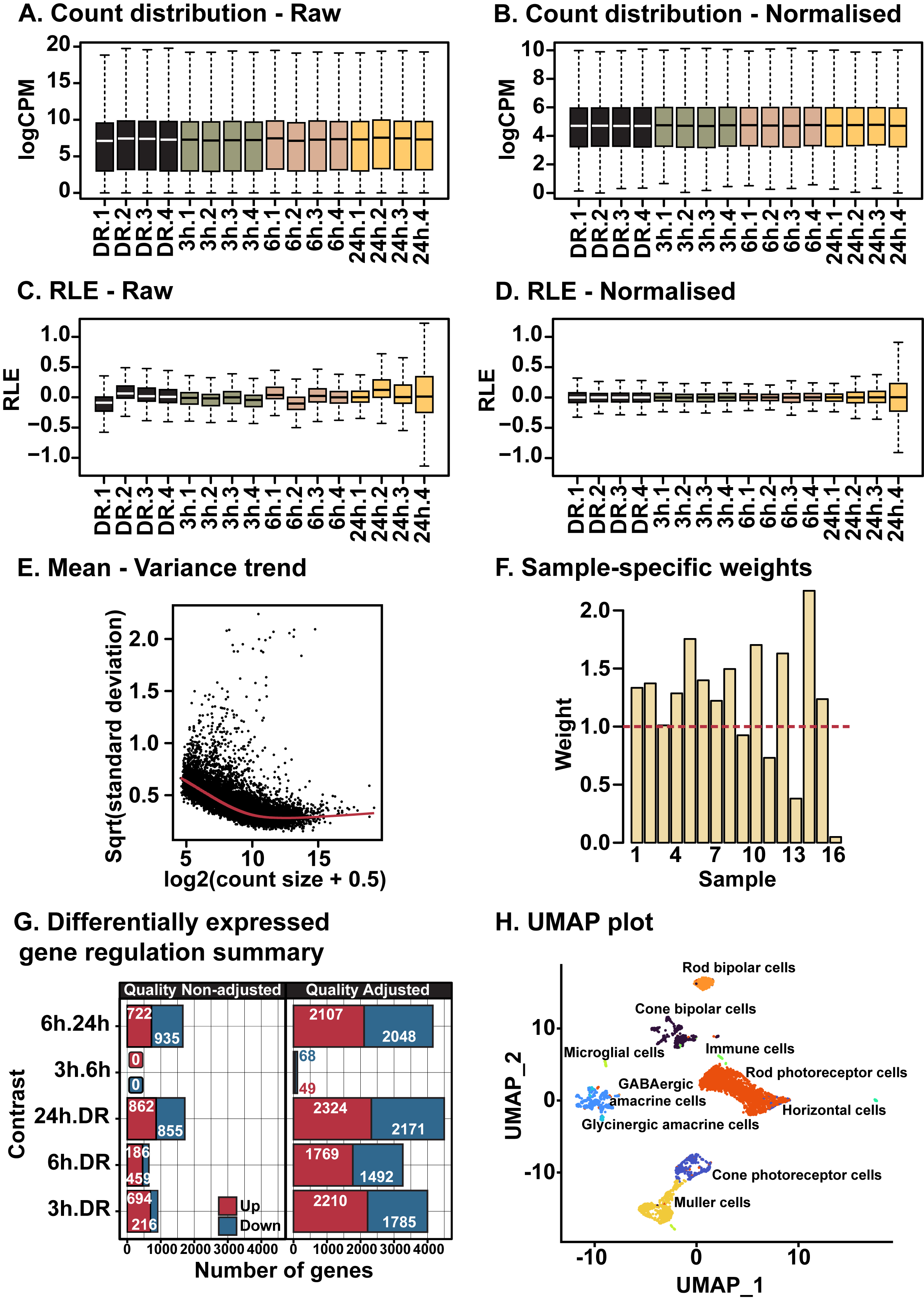

Supplement: Supplementary Figure 1 — Box plots of (A) raw and (B) normalised read count distributions showing effective normalisation (TMM). Relative log expression (RLE) box plots for (C) raw and (D) normalised counts. RLE values for each gene were calculated by deriving the median expression of each gene across all samples then computing the deviation from this median in each sample. (E) Mean variance relationship of each gene following voom transformation. (F) Summary of sample level variation obtained using voomWithQualityWeights function. (G) Summary of number of differentially expressed genes with and without adjusted for sample specific weights. As sample specific variation was observed, and correcting for unequal variation had a significant effect on the number of differentially expressed genes, the corrections for unequal variation were incorporated in the final lmFit statistical model. (H) Uniform manifold approximation projection plot (UMAP) of cell types obtained from the reanalysis of retinal single cell RNA sequencing data (74). [file Image_1.tif]

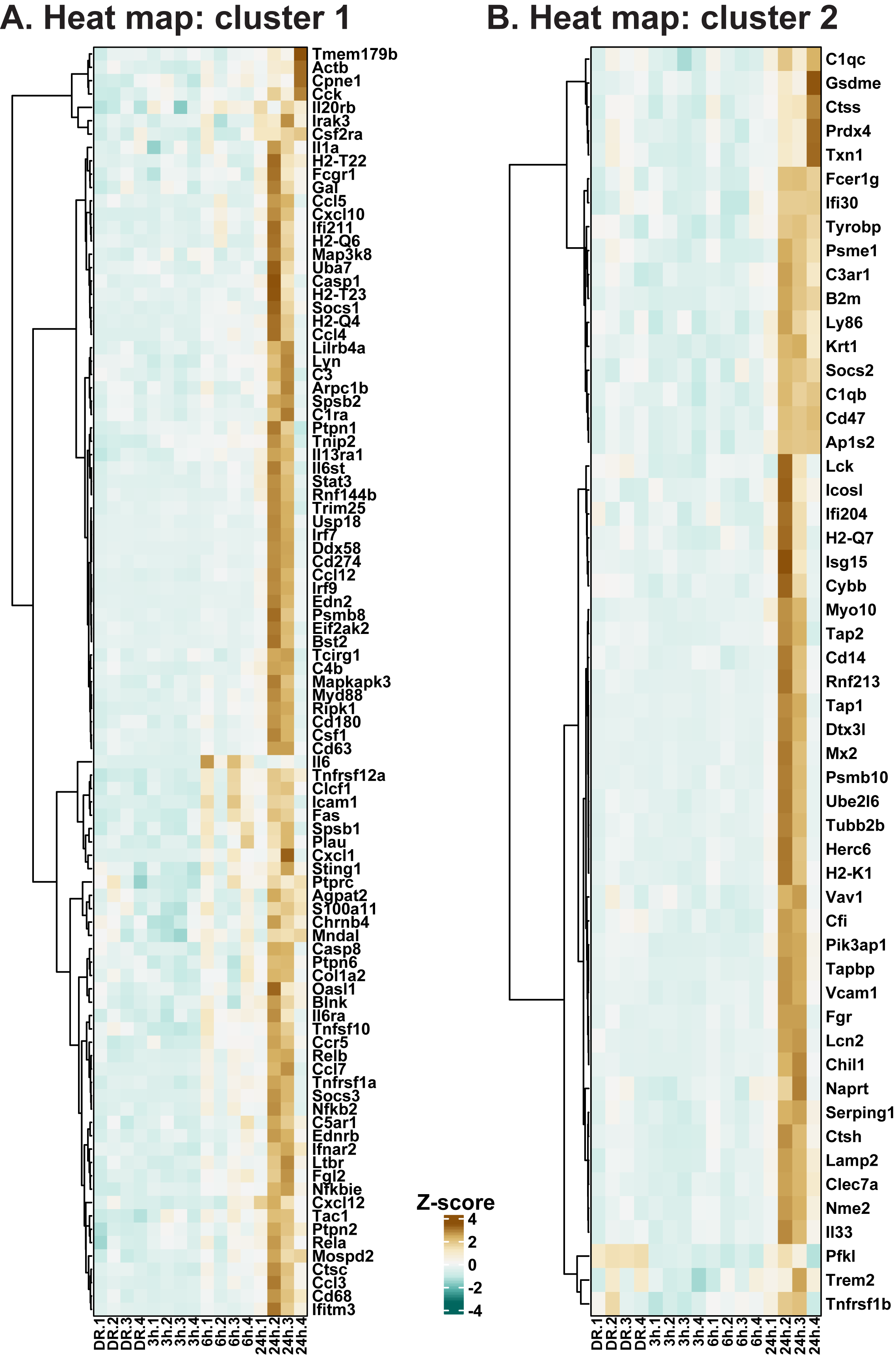

Supplement: Supplementary Figure 2 — Distinct gene profiles within dual inflammatory pathways in response to early photo-oxidative damage. (A, B) Heat maps show the expression profile of differentially expressed genes within cluster 1 and 2, with unique genes within each cluster. Significance P<0.05, n=4. [file Image_2.tif]
